# Understanding 3D Genome Organization and Its Effect on Transcriptional Gene Regulation Under Environmental Stress in Plant: A Chromatin Perspective

**DOI:** 10.3389/fcell.2021.774719

**Published:** 2021-12-08

**Authors:** Suresh Kumar, Simardeep Kaur, Karishma Seem, Santosh Kumar, Trilochan Mohapatra

**Affiliations:** ^1^ Division of Biochemistry, ICAR-Indian Agricultural Research Institute, New Delhi, India; ^2^ Decode Genomics Private Limited, New Delhi, India; ^3^ Indian Council of Agricultural Research, New Delhi, India

**Keywords:** Hi-C, ChIA-PET, single-cell 3D genomics, 4D genomics, chromosome territories, A/B compartment, topologically associating domain, chromatin loop

## Abstract

The genome of a eukaryotic organism is comprised of a supra-molecular complex of chromatin fibers and intricately folded three-dimensional (3D) structures. Chromosomal interactions and topological changes in response to the developmental and/or environmental stimuli affect gene expression. Chromatin architecture plays important roles in DNA replication, gene expression, and genome integrity. Higher-order chromatin organizations like chromosome territories (CTs), A/B compartments, topologically associating domains (TADs), and chromatin loops vary among cells, tissues, and species depending on the developmental stage and/or environmental conditions (4D genomics). Every chromosome occupies a separate territory in the interphase nucleus and forms the top layer of hierarchical structure (CTs) in most of the eukaryotes. While the A and B compartments are associated with active (euchromatic) and inactive (heterochromatic) chromatin, respectively, having well-defined genomic/epigenomic features, TADs are the structural units of chromatin. Chromatin architecture like TADs as well as the local interactions between promoter and regulatory elements correlates with the chromatin activity, which alters during environmental stresses due to relocalization of the architectural proteins. Moreover, chromatin looping brings the gene and regulatory elements in close proximity for interactions. The intricate relationship between nucleotide sequence and chromatin architecture requires a more comprehensive understanding to unravel the genome organization and genetic plasticity. During the last decade, advances in chromatin conformation capture techniques for unravelling 3D genome organizations have improved our understanding of genome biology. However, the recent advances, such as Hi-C and ChIA-PET, have substantially increased the resolution, throughput as well our interest in analysing genome organizations. The present review provides an overview of the historical and contemporary perspectives of chromosome conformation capture technologies, their applications in functional genomics, and the constraints in predicting 3D genome organization. We also discuss the future perspectives of understanding high-order chromatin organizations in deciphering transcriptional regulation of gene expression under environmental stress (4D genomics). These might help design the climate-smart crop to meet the ever-growing demands of food, feed, and fodder.

## 1 Introduction

A eukaryotic genome comprises several chromosomes, which vary along their length, contain supra-molecular complexes of chromatin fibers, and are intricately folded in a three-dimensional (3D) structure. The genome is not randomly positioned in the nucleus, but it is packed into higher-order chromatin structures that play important functional roles. Understanding the organization of the nuclear genome is seeking significant attention nowadays, as several processes like DNA replication, transcription, genome integrity, etc. involved in growth, development, and stress tolerance are regulated through the nuclear genome organization. Eukaryotic genome organization can be observed at three levels i) linear genome: the nucleotide sequence deciphered by DNA sequencing, ii) epigenome: representing the additional information added due to the modified bases and/or histone proteins which help regulate gene expression, and iii) 3D structure of the genome: representing the arrangement of chromatins/chromosomes in the nucleus (Bonev and Cavalli, 2016). These genome-level organizations are being studied with the help of recent advances in imaging and molecular biology techniques. To understand 3D genome structure, techniques like chromosome conformation capture (3C), chromosome conformation capture-on-chip (4C), chromosome conformation capture carbon copy (5C), chromatin interaction analysis by paired-end tag (ChIA-PET) sequencing, high-throughput chromosome conformation capture (Hi-C), and their derivatives are being used.

In eukaryotes, chromatin is packed into nucleosomes wherein histone proteins make up the largest component. DNA wrapped around a histone octamer (two units of each of the four core histones H2A, H2B, H3, and H4) sealed by a linker histone (H1) builds the structural constituent nucleosome to form chromatin. The chromatin-related research is progressing with unprecedented speed and resolution, deciphering the complex and dynamic chromatin architecture during cellular processes including DNA replication, recombination, repair, transcription, mitosis, and meiosis. Chromatin structures are highly dynamic, which undergo cyclic compaction and de-compaction during the cell cycle, cell differentiation, developmental processes, and defense responses. Chromatin accessibility to the regulatory elements like RNA polymerase II (RNA Pol-II) is affected by chromatin compaction/de-compaction, which fine-tunes the regulation of gene expression (Dixon et al., 2015; Boltsis et al., 2021). Differentiated cells have different cellular functions, and a different set of genes are expressed under different environmental conditions which require varying 3D genome architecture (Dixon et al., 2015). Changing environmental conditions (stresses) interfere with several cellular processes, which might require modulation in chromatin architecture to adjust the gene expression in response to the stress (Sun L. et al., 2020). Nucleotide sequence alone does not carry the entire regulatory information, as interactions among the chromosomes and topological changes in response to the developmental and/or environmental stimuli affect the expression of genes. Transient rearrangement of chromatin architecture (the compact heterochromatin or loosely-packed euchromatin) and modulation in chromatin composition upon stress exposure are being demonstrated in animals and plants (Lupianez et al., 2015; Li X. et al., 2019; Fei et al., 2019; Sun L. et al., 2020). Interaction of distal regulatory elements with the promoter through physical proximity mediated by the chromatin structural proteins like CCCTC-binding factor (CTCF) and cohesin to regulate the transcription process is being reported in animals (Eser et al., 2017; Robson et al., 2019).

Being sessile, plants face numerous abiotic and biotic stresses throughout their life. Our understanding of chromatin organization in model species has advanced significantly in the past decade (Sexton et al., 2012; Bonev and Cavalli, 2016; Grob and Grossniklaus, 2017). Highly condensed chromatin, such as heterochromatin, prevents accessibility of the transcriptional machinery (transcription factors, polymerases, and other nuclear proteins) to the gene. An environmental signal may cause some alterations in chromatin architecture which make the gene accessible to transcriptional machinery. Such chromatin remodeling includes shifting or removal of histones (Perrella et al., 2020), the introduction of histone variants (Dai et al., 2017; Wang et al., 2020), or post-translational modifications of histone proteins, etc. (Eberharter and Becker, 2002; Clapier et al., 2017). Studies show the hierarchical organization of genomes, wherein chromosome territories (CTs) are at the top of the hierarchical structure, followed by the chromosome compartments, topologically associating domains (TADs) and gene body/chromatin loops (Pecinka et al., 2004; Amano et al., 2009; Zhang and Wang, 2021). 3D genomics helps to decipher the spatial chromatin configurations and investigate their regulatory roles in gene expression (Gonzalez-Sandoval and Gasser, 2016).

Despite the absence of insulator protein CTCF in plants, TADs have rarely been observed in Arabidopsis. TAD-like domains and motifs at the TAD boundaries have been identified in rice (Liu et al., 2017). Moreover, cohesins subunits have also been identified in rice (Zhang et al., 2006; Tao et al., 2007; Gong et al., 2011). However, it is still not clear whether these cohesins have similar functions in plants. Inactive heterochromatic islands (IHIs) or KNOT engaged elements (KEEs) were reported to be present within euchromatin and exhibit strong long-range interactions in Arabidopsis (Feng et al., 2014; Grob et al., 2014; Grob and Grossniklaus, 2019), rice (Dong et al., 2018), and Brassica (Ting et al., 2019). Therefore, future investigations on the identification of CTCF-like insulator proteins, KNOT, KEEs and their functions in plants would be required.

Due to the sessile nature of plants, they deploy highly evolved mechanisms to manage their growth and development under varying environmental conditions (abiotic and biotic stresses). During the last few decades, linear genomes and epigenomes of eukaryotes have been extensively studied towards understanding the regulation of gene expression. It is now evident that the information and function of a genome are modulated under varying environmental conditions not only by the epigenetic modifications in the linear DNA sequence but also by altering the 3D chromatin organization within the nucleus (Dogan and Liu, 2018; Grob, 2020). Gene activities are controlled/regulated by alterations in chromatin architecture *via* DNA methylation (Kumar et al., 2018; Kumar and Mohapatra, 2021), histone modifications (Rowley et al., 2017; Park et al., 2018), and chromatin remodelers (Peterson and Workman, 2000; Bhadouriya et al., 2021). Different chromatin remodelers such as CHD, INO80, ISWI, and Switch/Sucrose non-fermenting (SWI/SNF) have been reported to act upon chromatin under diverse environmental stresses to convert transcriptionally inactive chromatin to the transcriptionally active state. Chromatin architecture at the promoter region is more crucial for determining the level of gene expression (Tannenbaum et al., 2018; Barragán-Rosillo et al., 2021). Advances in chromatin visualization, NGS, and 3C-based techniques have accumulated evidence for chromosome architecture, chromatin domains/loops and different epigenetic modifications to be correlated with transcriptional activities (Zhang and Wang, 2021). Studies suggest a functional correlation among the changes in nuclear organization, stressful conditions, and the level of gene expression. Tight coiling of chromatin (a default state) restricts transcriptional expression of the gene, which gets expressed when the nearby chromatin is loosened (remodeled) (Bannister and Kouzarides, 2011). The accumulating datasets on epigenomics (Kumar and Mohapatra, 2021) and the evident roles of genome architecture on the regulation of gene expression (Zhang and Wang, 2021) indicate that 3D genomics would be an important player in deciphering the key regulators. Developmental and environmental stimuli affect epigenetic landscape and chromatin architecture, which are dynamic and modulate gene expression to cope with stress (Bhadouriya et al., 2021). Some of the transcriptional repressors communicate with chromatin remodeler, directly or indirectly, or alter the chromatin structure. Some of these modifications may get transmitted through cell division, and help cope with the stress on reoccurrence (Gallusci et al., 2017). However, further validation of the transmission of stress-induced changes in chromatin architecture and their role in stress tolerance would be required.

This review presents the recent advances in 3D genomics methods and focuses on understanding the 3D genome organization of plants with reference to the available knowledge of nuclear genome organization in the animal system. We also discuss the developments in chromosome conformation capture technologies, their relevance in understanding genome structure (genome assembly) and functions. Future perspectives of 3D genomics, with special reference to its application in plant/crop improvement, and the constraints currently being faced are also discussed.

## 2 Understanding Nuclear Genome Organization

The eukaryotic genome is not randomly positioned in the nucleus, but it is packaged in a higher-order chromatin structure that plays important role in genome structure/functions. The spatial organization of chromatins allows an additional layer of regulatory information for transcriptional gene regulation that is far from the encoded information in the 1D genomic sequence (Lanctot et al., 2007). To explore the regulatory information of such organizational elements, the 3D structure of the genome has to be deciphered. Interacting nucleosomes make chromatin fiber, which physically interacts with the *cis*-acting elements to form chromatin loops (Crevillén et al., 2013). Structural proteins (CTCF, cohesin), transcription factors (TFs), and heterochromatin-binding proteins stabilize chromatin loops that form TADs (Wang Q. et al., 2018). TADs further interact to form chromatin compartments, which merge to constitute CTs (Lieberman-Aiden et al., 2009; Rao et al., 2014). Understanding 3D genome organization also demands to consider the sub-nuclear components like nuclear bodies (nucleolus, nuclear speckles, and Cajal bodies) and nuclear periphery (Mao et al., 2011; Rowley et al., 2017).

Genome organization is eminently dynamic, as it changes with the progression of the cell cycle, developmental transition (photomorphogenesis, flowering), and environmental cues (Kaiserli et al., 2018). In germinating Arabidopsis seedling, chromocenters were reported to be produced which could be visualized as large, bright spots on nuclear staining with DAPI (Bourbousse et al., 2015). Large chromatin regions associated with the nuclear periphery to form a network of lamina-associated domains (LADs), were reported in mammalian cells (Guelen et al., 2008). Some of the chromatin domains are also associated with the nucleolar periphery of nucleolus to form nucleolus-associated chromatin domains (NADs) (Nemeth et al., 2010; van Koningsbruggen et al., 2010). Although most of the 3D information on genome organization (e.g., LADs, NADs, TADs, etc.) in animals is comparable to that of plants, our knowledge of plant chromatin architecture is still in its infancy. Active and repressed chromatin regions are separated from each other in animals, and some of the nuclear compartments like nuclear-periphery and nucleolar-periphery are enriched with heterochromatin (repressed chromatin) (van Steensel and Belmont, 2017; Bersaglieri and Santoro, 2019). The chromatin domains localized at the nuclear/nucleolar periphery in Arabidopsis have been recently identified (Hu et al., 2019; Sun L. et al., 2020).

### 2.1 Deciphering 3D Genome Organization

Experimentation with 3D genome organization reveals that chromosomes occupy distinct nuclear spaces in the eukaryotic nucleus (Parada and Misteli, 2002). Additionally, chromosomes can show different configurations including Rab1, Rosette, and Bouquet configuration (Fransz et al., 2002; Harper et al., 2004; Grob and Grossniklaus, 2017). Advances in high-throughput 3C techniques and their derivatives help decipher the chromosomal interactions and address the complicated interplay between local chromatin organization and genome functions (Sexton and Cavalli, 2015). Individual chromosomes occupy separate CTs in the nucleus during interphase, which is the top hierarchical structure in most eukaryotic genomes (Pecinka et al., 2004). Further, chromosomes can be divided into A and B compartments wherein the A compartment is associated with high gene density/active transcriptional activity; the B compartment has higher transposon density and repressive epigenetic modifications. In mammals, TADs are enriched with chromatin loops on the hundreds of kilobase (Kb) scale which link promoters and *cis*-regulatory elements to modulate gene expression by recruiting TFs (Li et al., 2012; Rao et al., 2014; Rao et al., 2017). Moreover, compartmentalization within TAD protects the promoters from making ectopic contact with distant enhancers (Szabo et al., 2019). In contrast, the plant TADs might play different roles by making regulatory contact between enhancers and promoters occurring across TAD boundaries (Dong et al., 2017; Stam et al., 2019). Similarly, other structures like frequently interacting regions (FIREs), transcriptional hubs, and repressive loops have also been observed in plants while exploring the hierarchical chromatin interactions with the help of Hi-C (Dogan and Liu, 2018; Dong et al., 2018).

#### 2.1.1 Chromosome Territories

**FIGURE 1:**
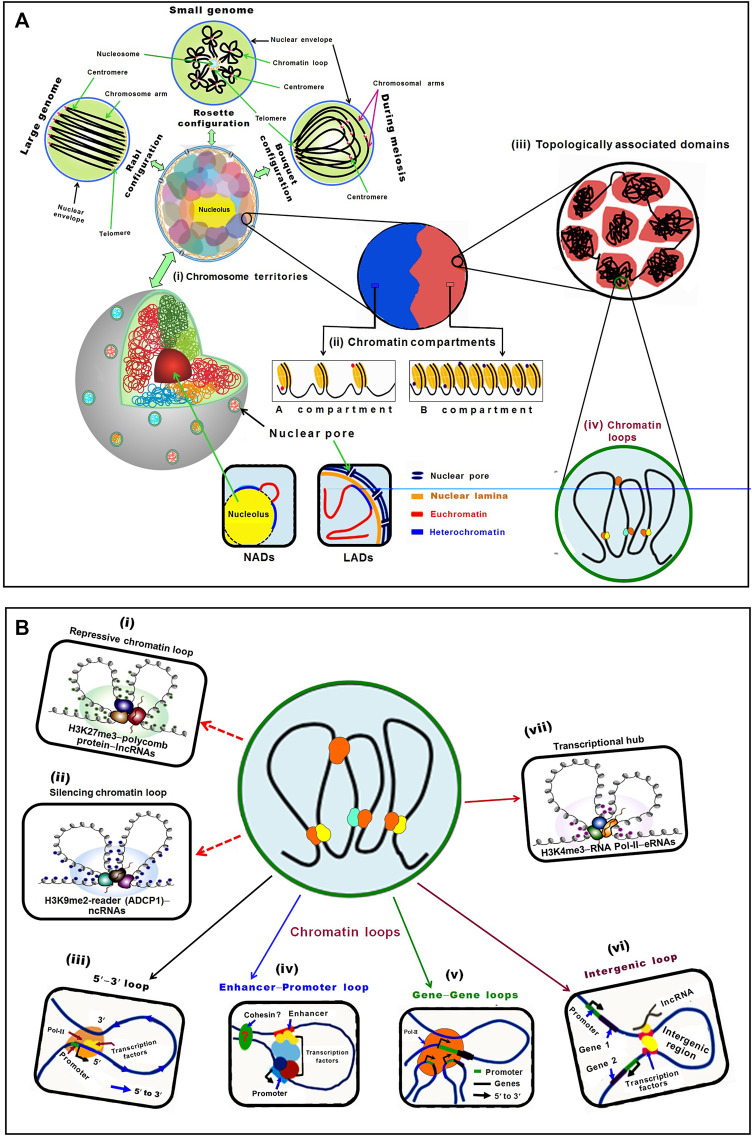
Schematic representation of plant chromatin organization in the nucleus. **(A)** Hierarchical chromatin organization can be studied mainly at four levels: chromosome territory, chromatin compartments, topologically associating domain (TAD), and chromatin loops. (i) Chromosomes occupy specific territories in the nucleus. In different territories, chromosomes show different morphology, such as Rabl, Rosette, and Bouquet configuration. In Rabl configuration, telomeres and centromeres of chromosomes cluster at two different poles in the nucleus, particularly in plants with larger genomes. In the Rosette configuration, the nucleolus is surrounded by telomeres, while heterochromatin and centromeres are clustered together but euchromatin oozes out freely in the nucleus to form a rosette-like configuration, observed in plants with smaller genome like Arabidopsis. Bouquet configuration is a transient chromatin configuration observed during meiosis in different organisms, including plants, wherein telomeres of the chromosomes are co-localized on a specific site of nuclear periphery, while the rest of the chromatin remains dispersed in the nuclear space. Nucleolus-associated domains (NADs) are chromatin regions that interact with the nucleolus, while the lamina-associated domains (LADs) are associated with the lamina of the nuclear envelope. Chromosome territories are further divided into (ii) A and B compartments, which correspond to euchromatic and heterochromatic regions, respectively. While the A compartment is constituted of high gene density, activating epigenetic modifications, and active transcriptional activity, the B compartment possesses lesser genes, low transcriptional activity, repressive epigenetic modifications, and higher transposon density. (iii) Topologically associated domains (TADs) are relatively independent local units/regions where chromatins interact with each other at a higher frequency than with the surrounding regions. (iv) Number of factors/modifications/readers is involved in the formation of chromatin loops that connects regulatory elements to their target loci in plants. **(B)** Lower level chromatin interactions (chromatin loops) establish regulatory networks between the distant elements through their physical proximity. The regulatory function of chromatin loops comes due to the formation of (i) heterochromatin/repressive loop by histone modifiers−H3K27me3−polycomb protein−lncRNAs, while (ii) silencing chromatin loop is formed by H3K9me2-reader (ADCP1)−ncRNAs. (iii) Different regions (5′–3′ gene looping) of the same gene, (iv) an enhancer and promoter (enhance–promoter loop) of a gene, (v) the different co-regulated genes (gene-gene loops), (vi) non-coding genomic regions (intergenic loop), and (vii) transcriptional hub/loop formed by H3K4me3 modifiers, RNA Pol-II and eRNAs.

Chromosome territory (CT) was directly visualized by cytological and microscopy techniques (Lichter et al., 1988; Pinkel et al., 1988). During mitosis interphase, each chromosome occupies an exclusive and limited domain in the nucleus called chromosome territory (CT) (Fransz and de Jong, 2011). Initial study on genome organization in Drosophila using Hi-C indicated sub-compartmentalization of chromosome arms (Sexton et al., 2012), and each chromosome occupies a space in the nucleus (Schubert et al., 2014) (Figure 1). CT is further subdivided into chromosomal arm territories, and a contact matrix of chromosome arms is more intricate than the contact matrix of the chromosome. Only weaker interaction in the pericentromeric regions, while strong interactions were reported between pericentromeric heterochromatin and telomeres in Arabidopsis (Grob et al., 2014). Studies on the genome of different crop plants (rice, maize, sorghum, tomato, and foxtail millet) revealed interactions between the adjacent loci and helped to understand the chromatin architecture (Dong et al., 2017). Intra- and inter-chromosomal interactions between the euchromatic arms resulted in the identification of CTs in all five plant species. While intense chromosomal interactions indicated frequent interactions between the chromosomal arms and centromeric regions of the chromosomes in the Hi-C map of maize, such interactions were not detected in rice and foxtail millet (Dong et al., 2020b). However, a signal for such interactions was reported in barley (Mascher et al., 2017).

In plants, chromosomes show different morphology including Rabl (in the honor of Carl Rabl), Rosette, and Bouquet configuration. In Rabl configuration, the chromosomes are folded at the centromere making a polarized separation of centromeres and telomeres (Figure 1A). Such configuration is observed in diverse organisms (animals, yeasts, and plants) (Huang Y. et al., 2020; Zhang and Wang, 2021). The existence of different chromosome configurations within an organism suggested its specificity in different cell types. The emergence of single-cell 3D genomics techniques would help to assess the linkage between chromatin organization and cell identity. In Rosette configuration, pericentromeric heterochromatin forms a condensed chromocenter from which euchromatic loops emerge out (Fransz et al., 2002). Traditionally, such configuration was attributed to the small genome of plant like Arabidopsis; however, having comparable genome size sorghum does not present this chromosome configuration (Muller et al., 2019). Moreover, yeasts having even smaller genomes than Arabidopsis presents Rabl configuration (Muller et al., 2019), indicating that genome size is not the determinant of chromosome configuration in the nucleus. In Bouquet conformation, telomeres cluster at the nuclear periphery while the chromatins emanate in the nucleoplasm (Figure 1A), which has been described in different plant species including rice, maize, and wheat during meiosis (Tiang et al., 2012; Zhang et al., 2017; Dogan and Liu, 2018; Hurel et al., 2018). Bouquet configuration appears to be a universal and transient feature of meiotic cells in plants, yeast, and animals (Huang Y. et al., 2020).

Recent studies reveal that gene expression is associated with chromatin positioning (CT) in the nucleus. Some of the chromatin domains are associated with the nucleolar periphery and named nucleolus-associated chromatin domains (NADs) (Pontvianne and Grob, 2020). In addition, the nuclear periphery is enriched with repressed chromatin associated with lamin fibers, and named as lamina-associated domains (LADs) (Gonzalez-Sandoval and Gasser, 2016). In plants, transcriptionally inactive LADs and NADs have been detected. In Arabidopsis, the LAD-specific protein, crowded nuclei 1 (CRWN1), has been reported to interact with polycombs1 (PWO1) to mediate chromatin tethering at the nuclear periphery (Poulet et al., 2017; Hu et al., 2019; Pontvianne and Liu, 2020). However, only limited research on LADs and NADs in plants have been carried out due to inadequate knowledge of the proteins required for the formation of these chromatin domains (Pontvianne and Liu 2020; Sakamoto et al., 2020).

#### 2.1.2 Global and Local A and B Compartments

Chromosomal compartments are formed due to the genome-wide interactions between TADs and epigenetic signatures, which have been discovered in both animals and plants while analysing Hi-C data. Two spatial compartments, namely A and B compartments, of chromosomes, have been reported. While A compartment is associated with open/active chromatin, the B compartment is associated with closed/inactive chromatin (Lieberman-Aiden et al., 2009; Dong et al., 2017) (Figure 1A). Apart from the A/B compartments, other compartment-like domains termed structural domains (SDs) have been reported in Arabidopsis. While the less compact euchromatin contains loose structural domains (LSDs), heterochromatin contains closed structural domains (CSDs) and is enriched in repressive epigenetic marks (Grob and Grossniklaus, 2017). The A compartment enriched with euchromatic activation histone marks, and the B compartment containing heterochromatic repressive epigenetic marks around the pericentromeric region have been reported in rice (Dong et al., 2018). Comparative analysis of rice, maize, and millet tissues using *in situ* Hi-C technique reported the existence of global A/B compartments across the tissues, while the local A/B compartment was reported to be dynamic and tissue-specific associated with differential expression of genes (Zhou et al., 2019; Dong et al., 2020a).

#### 2.1.3 Topologically Associating Domains (TADs)

In the eukaryotic genome, TADs are the independent local/structural units and the regions of high chromatin inter-connectivity. The A/B compartments can be further segmented as TADs which are 0.1–1.0 Mb in size (Dixon et al., 2012; Nora et al., 2012; Sexton et al., 2012; Rao et al., 2014). While the mammalian TADs are highly conserved in different tissues across the species (Dixon et al., 2012; Sexton and Cavalli, 2015; Vietri Rudan, et al., 2015; Grob and Grossniklaus, 2017), plant TADs are not conserved across the species (Dong et al., 2017). TADs are contiguous regions with more frequent chromatin interactions within the region than those with the other region in mammalian genomes (Dixon et al., 2012) (Figure 1A). TADs allow long-range chromatin interaction for target specificity of the remote *cis*-regulatory elements in plant and the human genome (Jin et al., 2013; Rao et al., 2014; Dogan and Liu, 2018). TADs are not reported in Arabidopsis because of the small genome size, as prominent TADs could not be detected in the species having smaller (<400 Mb) genomes (Dong et al., 2017; Stam et al., 2019). However, the effect of genome size on TAD formation is still under debate (Zhang and Wang, 2021). It is also speculated that TADs are displayed in plants having lower gene density/larger genome size (Dogan and Liu, 2018).

Animals TAD boundaries are reported to be bound by the insulator protein CCCTC-binding factor (CTCF) and specific epigenetic marks (Dixon et al., 2012; Sexton et al., 2012; Rao et al., 2014; Tang et al., 2015), which affect chromatin functions and transcriptional activity (Eser et al., 2017) through promoter-enhancer interactions. In plants, CTCF homologue has not been identified, which indicate that it might not be required for the formation of TAD boundary (Pontvianne and Grob, 2020). Growing evidence suggests that cohesin couple with CTCF in TAD establishment in mammals (Fudenberg et al., 2016; Rao et al., 2017; Nuebler et al., 2018). Cohesins are conserved between animals and plants (Zhang et al., 2004); however, the cohesins have similar functions in plants is still not clear (Ouyang et al., 2020). It would be interesting to identify CTCF-like insulator proteins in plants involved in the formation of TAD boundaries.

In embryonic stem cells of mice, high-resolution (allele-specific 4C) mapping indicated that TAD is constituted of metaTADs and subTADs, which are dynamic to form active and inactive nuclear compartments (Wijchers et al., 2016). Wijchers et al. (2016) suggested that *trans*-associated factors (SUV39H1, or EZH2) influence 3D compartmentalization independent of their *cis*-effect on local chromatin composition and activity. In Arabidopsis, several local structural features like positive strips which interact frequently with the neighboring chromatin were observed (Wang et al., 2015). Such positive strips enriched with repressive histone marks like H3K27me3 were reported in Arabidopsis (Liu Z. et al., 2016). In plants, TAD-like domains lack co-expression behavior and do not possess a conserved biological function as observed in mammals (Dekker and Heard, 2015). Moreover, the TADs rich in GC motifs and positively correlated with transcriptional activation/gene expression were observed in rice and cotton (Liu et al., 2017).

Similarly, TAD-like domains enriched in and associated with highly expressed genes were observed in maize, tomato, foxtail millet, and sorghum (Dong et al., 2017). Hi-C analysis of diploid and tetraploid cotton suggested the existence of intra-chromosomal interactions and TAD-like regions (Wang M. et al., 2018). In rice, TADs showed increased sequence variation and meiotic recombination compared to that observed in the inter-TAD regions (Golicz et al., 2020). In wheat, the existence of TAD-like structures (termed as intergenic condensed spacers, ICONS) was reported (Concia et al., 2020). Therefore, it would be interesting to examine whether the occurrence of TADs/TAD-like structures is linked with larger genome size. In general, these observations support the hypothesis that plant genomes are packaged into TAD-like structures by yet to be identified molecular mechanism(s). The *cis*-regulatory elements of target genes form chromatin loops to control gene expression.

#### 2.1.4 Chromatin Loops

Another level of hierarchical genome organization that plays important role in transcriptional regulation of gene expression is the chromatin loop. The chromatin loops are formed due to physical interaction between *cis*-acting elements and the genes that are brought into close spatial proximity, which are vital for gene regulation (Bonev and Cavalli, 2016) (Figure 1B). In plants, chromatin loops are formed between distal regulatory elements and promoters to exert their function by providing the opportunity for enhancers to contact with their genes located at tens of kilobase-pair away (Dogan and Liu, 2018; Li E. et al., 2019). In maize, the first chromatin loop was observed between the promoter and regulatory sequences at *b1* locus (Louwers et al., 2009), and it was reported between 5′ and 3′ flanking regions of flowering locus C (FLC) in Arabidopsis (Crevillén et al., 2013). Moreover, chromatin loops of varying sizes (Kb to Mb, small as well as large loops) have been reported in the eukaryotic genome (Rao et al., 2014). Generally, transcription start site loops with the downstream region and transcription termination site loops with the upstream region. The formation of such loops enhances promoter−enhancer interaction to initiate the transcription process. Loop structure has also been reported in the formation of rosette-like structure in the heavy chain of immunoglobulin that is required for V(D)J recombination (Ebert et al., 2015). *In situ* Hi-C analysis revealed extensive chromatin loops in the regions enriched with epigenetic marks and active genes in the larger genomes like maize and tomato, while such loops are absent in the smaller genome (Dong et al., 2017). Spatial organization of the regulatory elements revealed by the construction of high-resolution chromatin interaction maps in maize deciphered the role of chromatin loops in gene expression (Peng et al., 2019). The active and repressed chromatin regions are separated from each other in animals, and some compartments in the nucleus, like nuclear and nucleolar periphery, are enriched with repressed chromatin (van Steensel and Belmont, 2017; Bersaglieri and Santoro, 2019).

Histone modifiers (e.g., H3K9me reader ADCP1) and non-coding RNAs (ncRNAs) are involved in the formations of repressive chromatin loop [Figure 1B(i)], while H3K27me3 and long non-coding RNAs (lncRNAs) are associated with silenced chromatin loop formation [Figure 1B (ii)] (Stam et al., 2019; Kantidze and Razin, 2020). The 5′−3′ loop bring together the 5′ and 3′ termini of the same gene [Figure 1B (iii)], an enhancer-promoter loop occurs between the promoter and distant enhancer of a gene [Figure 1B (iv)], gene—loop is formed between different co-regulated genes [Figure 1B (v)], an intergenic loop is comprised of the intergenic region (Rodriguez-Granados et al., 2016; Huang Y. et al., 2020) [Figure 1B (vi)], and transcriptional hub is formed by certain activation histone marks/modifiers (e.g., readers, writers, mediators of H3K4me3), RNA Pol-II, and RNAs (Ouyang et al., 2020) [Figure 1B (vii)].

RNAs and multivalent proteins play vital roles in the formation of chromatin loops. Enhancer RNAs (eRNAs) combined with mediator and RNA Pol-II were reported to promote the formation of enhancer-promoter loops to modulate transcriptional activity of the target genes in human cell lines (Lai et al., 2013; Pefanis et al., 2015) [Figure 1B (vii)]. In Arabidopsis, a Mediator subunit (MED25) was reported to affect the dynamics of chromatin looping between the promoter and enhancer to trigger transcriptional programming in the jasmonic acid signaling pathway (Wang et al., 2019). Activating histone marks (H3K4me3 and H3K36me3) and histone variants (H2A.Z, and H2Bub1) were observed at the *FLC* locus which is bound by histone modifiers like H3K4 methyltransferase and H3K36 methyltransferase (Li Z. et al., 2018). Binding of these ‘writers’ to *FLC* results in the formation of 5′-to-3′ loop.

## 3 3D Genome Mapping Techniques

**FIGURE 2:**
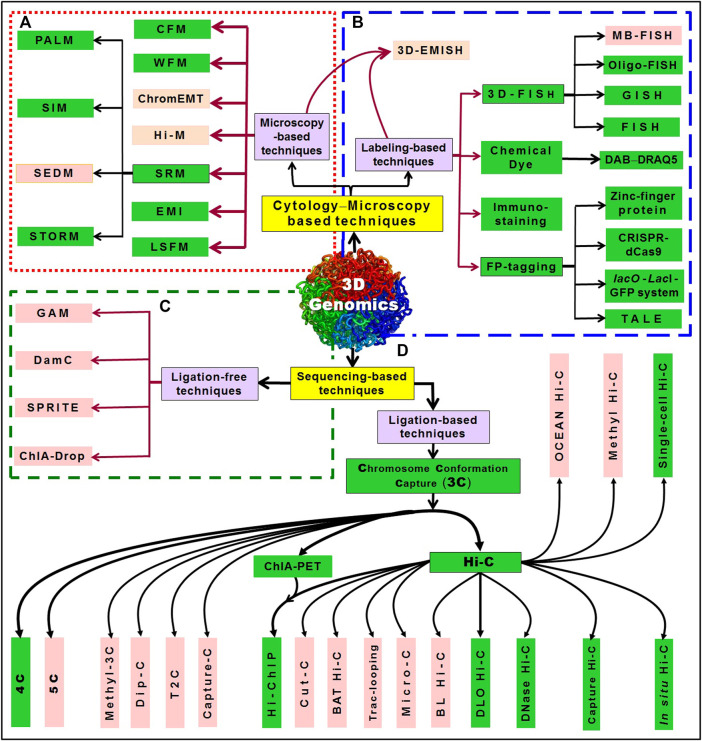
An overview of 3D genomics techniques. The techniques can be broadly divided into two categories: one is based on cytological/microscopic examination/imaging; another is based on sequencing. **(A)** The microscopy-based techniques include WFM (wide-field microscopy), CFM (confocal fluorescence microscopy), ChromEMT (Chrom-electron microscopy tomography), Hi-M (multiplexed, sequential imaging approach) simultaneously reveals 3D chromatin organization and transcriptional activity, SRM (super-resolution microscopy), which include SIM (structured illumination microscopy), SEDM (stimulated emission depletion microscopy), PALM (photoactivated localization microscopy), STORM (stochastic optical reconstruction microscopy), EMI (electron microscopy imaging), and LSFM (light-sheet fluorescence microscopy). **(B)** While 3D-EMISH (three-dimensional electron microscopy with *in situ* hybridization) utilizes the advantages of both microscopy (electron microscopy) and labeling (*in situ* hybridization), the labeling-based techniques include 3D-FISH [such as fluorescence *in situ* hybridization, MB-FISH (molecular beacon-FISH), Oligo-FISH (oligonucleotides probe-based FISH), GISH (genomic *in situ* hybridization)], staining with chemical dyes like DAB−DRAQ5 system, immune-staining, and FP-tagging (fluorescent protein-tagging) including tagging with zinc-finger protein, *lacO*-LacI-GFP system, CRISPR-dCas9 (clustered regularly interspaced short palindromic repeats-nuclease-deficient Cas9), TALE (transcription activator-like effectors with a quantum dot labeling technique). The sequencing-based techniques can be ligation-free or those which require proximity ligation (3C, chromosome conformation capture). **(C)** While ligation-free techniques include GAM (genome architecture mapping) that combines micro-cutting and sequencing, SPRITE (split-pool recognition of interactions by tag extension), ChIA-Drop (chromatin interaction analysis *via* droplet-based and barcode-linked sequencing), DamC (DNA adenine methyltransferase-based chromosomal contacts), **(D)** ligation-based techniques include the advancements in 3C (chromosome conformation capture), like 4C, 5C (chromosome conformation capture carbon copy), methyl-3C (combination of DNA methylation detection and 3C technology), Dip-C (combination of single-cell 3C and transposon-based whole-genome amplification method), T2C (targeted Chromatin Capture), Capture 3C (combination of 3C with oligonucleotide capture technology. Further advancements like ChIA-PET (chromatin interaction analysis by paired-end tag sequencing) and Hi-C (high-throughput chromosome conformation capture), and their combination Hi-ChIP (chromatin conformation method that combines Hi-C with ChIA-PET technology) have advanced the 3D genome architectures. Combination of techniques like Cut-C (antibody-mediated cleavage by tethered nuclease with chromosome conformation capture), Capture Hi-C (combination of Hi-C and hybridization-based capture of targeted genomic regions), *in situ* Hi-C (DNA–DNA proximity ligation performed in intact nuclei), Micro-C (chromatin fragmented into mononucleosomes using micrococcal nuclease), DNase Hi-C (chromatin fragmented by DNase I), DLO Hi-C (digestion-ligation-only Hi-C), BAT Hi-C (bridge linker-*Alu*l-Tn5 Hi-C), BL Hi-C (bridge linker Hi-C), Trac-looping (transposase-mediated analysis of chromatin looping), Methyl Hi-C (a combination of DNA methylation detection technology and Hi-C), OCEAN Hi-C (open chromatin enrichment and network Hi-C), and Single-cell Hi-C (Hi-C in an individual nucleus). The techniques that have been successfully used in plants are presented in the green box (modified from Pei et al., 2021).

The advances/improvements in bioimaging and biochemical methods over the last few decades have unveiled 3D genome architectures in animals and plants at a rapid speed (Sexton and Cavalli, 2015; Ouyang et al., 2020). The 3D genome mapping approaches can be broadly divided into two categories. The first category of approaches is based on cytology/microscopy, which utilizes fluorescent dye to label DNA/chromatin and/or visualization of the spatial chromatin organization using a microscope (Probst, 2018). Combining microscopy with fluorescent *in situ* hybridization (FISH) boosted the progress in understanding how the spatial organization of CTs affects gene expression within the nucleus (Zhang and Wang, 2021). The second group of approaches utilizes next-generation sequencing (NGS), and they could be ligation-based or ligation-free (Figure 2). Each of these techniques has certain advantage over the other, and there is some limitation in using then individually in 3D genome analysis (Table 1). However, a combination of techniques provides better opportunity for improved specificity, sensitivity, and ultrahigh-resolution analysis.

**TABLE 1:** Characteristic features of different techniques used for 3D genome organization analysis.

Technique	3D genomics approach	Advantage	Limitation	References
Microscopy-based techniques	Visualize chromatin conformation by cytological and microscopy, indispensable for single-cell genome organization studies	Cytological expertise can be exploited for more efficient analysis, may simultaneously analyze 3D chromatin organization and transcriptional activity	Limited resolution of the traditional microscopic technique needs to combine with other technique to improve the resolution	Lobastov et al. (2005); Wheeler and Tyler (2011); Cardozo Gizzi et al. (2019)
Labeling-based techniques	Label DNA/chromatin to visualize the spatial chromatin organization	Improve sensitivity, specificity and resolution; enable the possibility for live-cell imaging	Repetitive sequence required for easy visualization; stringent preparation/protocol	Schubert et al. (2001); Matzke et al. (2005); Wu et al. (2010); Ma et al. (2017); Nagaki and Yamaji (2020)
Ligation-free techniques	Do not require proximity-ligation but use sequencing technologies for in-depth chromosomal interaction analysis	Can detect distal chromatin interaction along with the methylation status, also detect DNA−RNA interactions	Pairwise interaction between two loci	Beagrie et al. (2017); Quinodoz et al. (2018); Redolfi et al. (2019); Zheng et al. (2019)
*C*hromosome *C*onformation *C*apture (3C)	Rely on enzymatic digestion of DNA followed by proximity ligation to capture long-range chromatin interaction between two specific genomic loci	Captures long-range chromatin interaction between two specific genomic loci	Low throughput coverage, provides chromatin configuration of population average, presents one-to-one interaction	Dekker et al. (2002)
*C*hromosome *C*onformation *C*apture-on-*C*hip (4C)	Circular chromosome conformation capture approach, detected by inverse-PCR using the primers for candidate gene	Studies the interaction between a chromatin site of interest and the other sites on whole genome	Less efficient to study the interactions of shorter distance (<50 Kb); reveals one-to-many interactions	Simonis et al. (2006); Zhao et al. (2006); Grob and Cavalli (2018)
*C*hromosome *C*onformation *C*apture *C*arbon *C*opy (5C)	Analyses the interactions with in a limited region like gene clusters, templates originating from the region of interest are PCR amplified and quantified using NGS approach	Used for chromatin interaction analysis between multiple genomic loci (many-to-many interactions); bioinformatics play important role in the analysis	Suitable for interaction studies on relatively smaller genomes only	Dostie et al. (2006); Sati and Cavalli (2017)
ChIA-PET	DNA–protein complex is cross-linked, fragmented by ultrasonication, and captured by the protein-specific antibody (ChIP) which is analysed by high-throughput sequencing	Efficient analysis of long-range chromatin contacts bound by a protein and provides a high-resolution map of chromatin interactions with considerably fewer sequencing reads	Captures the distal interactions where specific proteins are involved; hromatin configuration of population average; reveals many-to-many interactions	Fullwood et al., 2009; Li et al., 2010; Li et al., 2017
Hi-C	Relies on restriction enzyme to break the chromatin into smaller fragments, uses NGS approach to investigate both short- and long-range chromatin interactions at whole-genome level	Detects “all-to-all” interactions	May not be appropriate for the study of individual locus; generates abundant unenriched chromatin contact data	Lieberman-Aiden et al. (2009); Feng et al. (2014); Wang et al. (2017); Xie et al. (2019)
Hi-ChIP	A protein-centric chromatin conformation method, Hi-C is combined with ChIA-PET.	Ten-fold more informative reads with 100 times lesser input requirement; generates significantly better signal-to-noise ratio	The protein-specific antibody is required to capture the DNA–protein interactions; produces unenriched chromatin contact data	Mumbach et al. (2016); Ricci et al. (2019); Concia et al. (2020)
BAT-Hi-C	Combines *Alu*l restriction with biotinylated linker-mediated proximity ligation analysis	Ideal for genome-wide in-depth analysis of long-range chromatin looping; economical and straightforward technique	Chromatin configuration of population average; need optimization for plant studies	Huang et al. (2020a)
Capture Hi-C	Combines Hi-C and hybridization-based capture of targeted genomic regions	Specific probes are used to capture the reads related to the target region, and chromatin interactions of the region are deciphered by NGS.	Chromatin configuration of population average	Mifsud et al. (2015)
*In situ* Hi-C	The intact nuclei are used, instead of free chromosomes, for ligation	Use of complete nuclei reduces wrong ligation of DNA fragments from different nuclei, effectively reduce the background noise, and improving the signal-to-noise ratio	Chromatin configuration of population average	Rao et al. (2014); Liu et al. (2017)
Methyl Hi-C	Combination of Hi-C and DNA methylation detection technology	Simultaneous captures the chromosome conformation and DNA methylation	Chromatin configuration of population average	Li et al. (2019)
Single-cell Hi-C	Chromatin conformation of individual cell is captured and studied using Hi-C at single-cell level	Chromatin conformation of an individual cell is captured; avoids averaging of chromatin maps for a population of cells	Still in its infancy for plant studies	Nagano et al. (2013); Stevens et al. (2017); Zhou et al. (2019); Sun et al. (2021)

The cytology/microscopy-based approaches can be further divided into either microscopy or labeling-based techniques. For single-cell genome organization study, microscopy is indispensable. Although fluorescence microscopy has enabled us to investigate larger chromosome organizations of micron length scale, the smaller structures remained invisible due to the limited spatial resolution of fluorescence microscopy (Lakadamyali and Cosma, 2015). However, the advent of super-resolution microscopy (SRM) enables us to investigate nano-scale chromosome organizations *in vivo*. Such SRM methods have the potential to enhance our knowledge of chromatin structure−function relationship. Microscopy-based techniques include confocal microscopy (CFM) (Carlsson et al., 1989), wide-field microscopy (WFM) (Wheeler and Tyler, 2011), chrom-electron microscopy tomography (ChromEMT) *in situ* visualization of chromatin using a fluorescent dye that stains DNA with an osmiophilic polymer with selectively enhances the contrast in electron microscopy (Ou et al., 2017), Hi-M (a multiplexed, sequential imaging approach) simultaneously reveals 3D chromatin organization and transcriptional activity; thus, enables detecting the spatial organization of cells and measurement of the changes in TAD organization during early embryogenesis and upon transcriptional activation (Cardozo Gizzi et al., 2019), SRM (Schubert, 2017), electron microscopy imaging (EMI) (Lobastov et al., 2005), and light-sheet fluorescence microscopy (LSFM) (Santi, 2011). SRM depends on photoactivated localization microscopy (PALM) (Rust et al., 2006), structured illumination microscopy (SIM) (Fitzgibbon et al., 2010), stimulated emission depletion microscopy (SEDM) (Dyba et al., 2003), stochastic optical reconstruction microscopy (STORM) (Betzig et al., 2006) (Figure 2A). Intensive research on microscopic visualization of chromosome organization using labeling-based techniques like FISH has greatly improved the sensitivity, specificity and resolution (Cui et al., 2016). Combining FISH and super-resolution microscopy further boost the detailed characterization of structural chromatin domains (Boettiger et al., 2016). 3D-EMISH (combines serial block-face scanning electron microscopy with *in situ* hybridization) visualizes 3D chromatin folding at targeted genomic regions with ultrahigh-resolution (5 nm × 5 nm × 30 nm) (Trzaskoma et al., 2020). Depending on the type of label/dye used, the labeling techniques are divided into four categories: i) 3D-FISH, which include fluorescence *in situ* hybridization (FISH) (Solovei et al., 2002; Koornneef et al., 2003; Berr and Schubert, 2007; Cremer et al., 2008); Oligo-FISH, uses oligonucleotide probes (Beliveau et al., 2012; Beliveau et al., 2012); MB-FISH, uses molecular beacon probes (Wu et al., 2010; Ni et al., 2017); GISH, genomic *in situ* hybridization (Schubert et al., 2012), ii) staining with chemical dyes, e.g., DAB−DRAQ5 system, (Ou et al., 2017; Poulet et al., 2017), iii) immuno-staining (Fransz et al., 2002; She et al., 2013), and iv) fluorescent protein-tagging (FP-tagging) (Matzke et al., 2005; Lindhout et al., 2007; Deng et al., 2015; Ren et al., 2017; Nagaki and Yamaji, 2020) which include tagging with zinc-finger proteins, clustered regularly interspaced short palindromic repeats−nuclease-deficient Cas9 (CRISPR-dCas9) (Dreissig et al., 2017; Hong et al., 2018), *lacO*-LacI-GFP system (Ding and Hiraoka, 2017), and transcription activator-like effectors (TALE) coupled with quantum dot labelling technique (Ma et al., 2017). Chromatin domain can be labeled using dCRISPR-Cas9 reporter proteins (guided by sgRNA) or green fluorescent protein-tagged m6A-tracer protein, which allow tracking the location of chromatin domain in the nucleus (Qin et al., 2017; Ye et al., 2017; Hong et al., 2018; Wong et al., 2021). The *lacO*/LacI-GFP system provides a simple and useful method to visualize a chromosome locus by inserting *lacO* repeat arrays and expressing LacI–GFP fusion protein that binds to the *lacO* (Ding and Hiraoka, 2017). A novel bimolecular fluorescence complementation (BIFC) method, that combines the advantages of both dCas9-labeling and gRNA-labeling, enables live cell imaging with high signal-to-noise ratios without non-specific foci (Hong et al., 2018) (Figure 2B).

Some of the sequencing-based techniques that do not require proximity-ligation have also been devised for 3D genomics studies, which include genome architecture mapping (GAM) (Beagrie et al., 2017), split-pool recognition of interactions by tag extension (SPRITE) (Quinodoz et al., 2018), DNA adenine methyltransferase identification of chromosomal interactions (DamC) (Redolfi et al., 2019), and chromatin interaction analysis *via* droplet-based and barcode-linked sequencing (ChIA-Drop) (Zheng et al., 2019) (Figure 2C). While GAM utilizes micro-sectioning and sequencing to decipher the relative location of genes and enhancers for studying the frequency of genomic interactions in nuclear sections (Beagrie et al., 2017), SPRITE detects pairwise interactions between two loci as well as DNA−RNA interactions (Quinodoz et al., 2018), DamC detects distal chromatin interaction along with the methylation status wherein DNA adenine methyltransferase and DNA-binding proteins are recruited to specific genomic locations (Redolfi et al., 2019). On the other hand, ChIA-Drop uses a specific antibody to capture the target protein and interacting DNA by ChIP for multiplex chromatin-interaction analysis adopting microfluidics to produce gel-bead-in-emulsion droplets (Zheng et al., 2019).

Another group of techniques that use proximity-ligation and NGS includes chromosome conformation capture (3C)-based approaches (Dekker et al., 2002) as well as its derivatives such as chromosome conformation capture-on-chip (4C) (Simonis et al., 2006), chromosome conformation capture carbon copy (5C) (Dostie et al., 2006) (Figure 2D). While 3C-based techniques rely on enzymatic digestion of DNA and proximity ligation to capture long-range chromatin interaction between two specific genomic loci (Dekker et al., 2002), 4C is used to visualize the interaction between a site of interest and other sites on the genome (Simonis et al., 2006). Moreover, 5C is used to analyze the chromatin interactions between multiple genomic loci (Dostie et al., 2006). A combination of DNA methylation detection and 3C technology (methyl-3C) (Lee et al., 2019), a combination of single-cell 3C and transposon-based whole-genome amplification (Dip-C) (Tan et al., 2018), targeted chromatin capture (T2C) studies chromatin organization for specific genomic regions (Kolovos et al., 2014), while capture-C combines 3C with oligonucleotide capture technology (Hughes et al., 2014). Further developments in 3C sequencing technologies resulted in Hi-C and ChIA-PET which have been quite helpful in 3D genomic studies (Fullwood et al., 2009; Lieberman-Aiden et al., 2009). A protein-centric chromatin conformation method that combines Hi-C with ChIA-PET technology (Hi-ChIP) (Mumbach et al., 2016) has improved our understanding of 3D genome architectures. Hi-ChIP improves the informative reads by over 10-fold and lowers input requirement by over 100-fold compared to ChIA-PET. Being an efficient and sensitive analysis of protein-directed genome architecture, Hi-ChIP for cohesin reveals multi-scale genome architecture with greater signal to the background than *in situ* Hi-C (Mumbach et al., 2016). Capture Hi-C combines Hi-C and hybridization-based capture of targeted genomic regions (Mifsud et al., 2015), *in situ* Hi-C is performed in the intact nuclei with DNA–DNA proximity-ligation (Rao et al., 2014), micro-C uses micrococcal nuclease for chromatin fragmentation (Hsieh et al., 2015), while DNase Hi-C uses DNase I enzyme to fragment the chromatin (Ma et al., 2015) for chromatin architecture analysis. Similarly, single-cell Hi-C (Hi-C analysis of nucleus from a single-cell, Nagano et al., 2013) bridges the gap between genomics and microscopy studies of chromosome structure, the *B*ridge *L*inker Hi-C (BL Hi-C) combines restriction enzyme (RE) targeting and two-step proximity ligation (Liang et al., 2017), while in *D*igestion-*L*igation-*O*nly Hi-C (DLO Hi-C) digestion and ligation are performed twice without biotin labeling and pulldown (Lin et al., 2018). The recently developed techniques like Trac-looping (transposase-mediated analysis of chromatin looping) for simultaneous detection of multiscale genome-wide chromatin interactions among regulatory elements and chromatin accessibility (Lai et al., 2018), and OCEAN Hi-C (open chromatin enrichment and network Hi-C) for antibody-independent mapping of global open chromatin interactions (Li T. et al., 2018) were used to decipher the chromatin architecture. More recently, Cut-C combined antibody-mediated cleavage by tethered nuclease with chromosome conformation capture to identify chromatin interactions mediated by a protein of interest (Shimbo et al., 2019). Cut-C identifies protein-centric chromatin conformations along with the genome-wide distribution of target proteins using a simple procedure. Applying Cut-C to a histone modification (H3K4me3) enriched at active gene promoters, Shimbo et al. (2019) could successfully identify the chromatin loops mediated by H3K4me3 along with the genome-wide distribution of H3K4me3. Further, methyl Hi-C (DNA methylation detection combined with Hi-C) for simultaneous capture of chromosome conformation and DNA methylome was used to delineate the DNA methylation profile and chromatin architecture of a cell (Li G. et al., 2019). A simple technique for economical but efficient analysis of chromatin conformational features in mouse embryonic stem cells, BAT Hi-C (*B*ridge linker-*A*lul-*T*n5 Hi-C), was developed by combining *Alu*l restriction with biotinylated linker-mediated proximity ligation (Huang J. et al., 2020). With just one-third sequencing depth, BAT Hi-C could reveal the same spectrum of chromatin contacts as *in situ* Hi-C. Being an economical and straightforward technique, BAT Hi-C is ideal for genome-wide in-depth analysis of long-range chromatin looping (Huang J. et al., 2020). While many of these techniques have been successfully used in the animal system, some of them need to be optimized in plants as efficient/economical and simple techniques (Figure 2).

### 3.1 Chromosome Conformation Capture (3C) and Its Derivatives

**FIGURE 3:**
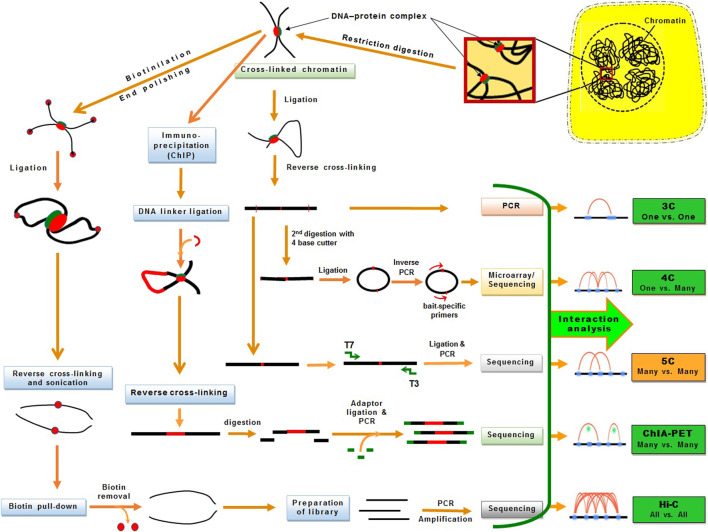
An overview of the chromatin conformation capture (3C) and its derivative techniques used for 3D genomic studies. The DNA−protein interactions are fixed *in vivo* using formaldehyde and then chromatins are fragmented by restriction endonuclease treatment. The cross-linked chromatins are processed differentially for one vs one (3C), one vs many (4C), many vs many (5C, ChIA-PET), or all vs all (Hi-C) interaction analysis. The techniques successfully used in plants are presented in green boxes.

The 3D genomic techniques have considerably advanced over the last decade. However, their efficiency in plant 3D genomic studies is comparatively less probably because of the cell wall. Some of the derivative techniques have successfully been used in plants for 3D genomic studies. The basic 3C technique allows “one-to-one” chromosomal interactions between two loci in the genome utilizing microarrays. Hence, the 4C technique was developed as a “one to all” strategy, which allows genome-wide screening for the interactions between one specific locus with all other loci in the genome (Zhao et al., 2006) using NGS to determine long-range chromatin interactions (Splinter et al., 2012). Since 4C is suitable for long-range interaction studies (Grob and Cavalli, 2018), the 5C technique was developed for the detection of “many to many” chromosomal interactions among thousands of selected genomic loci in a single run (Dostie et al., 2006; Simonis et al., 2007) (Figure 3).

Later, the Hi-C technique was devised for detecting “all-to-all” interactions between any locus with all other chromosomal loci with far-reaching impacts (Lieberman-Aiden et al., 2009). Hi-C uses high-throughput sequencing to investigate both short- and long-range chromatin interactions at the whole-genome level (Lieberman-Aiden et al., 2009). Hence, Hi-C has been extensively used for characterizing chromosomal architecture in plant species like Arabidopsis, rice, tomato, Brassica, cotton, foxtail millet, sorghum, and maize (Feng et al., 2014; Grob et al., 2014; Wang et al., 2015; Wang et al., 2017; Dong et al., 2018; Grob and Cavalli, 2018; Sotelo-Silveira et al., 2018; Wang M. et al., 2018; Ting et al., 2019; Xie et al., 2019) (Figure 3). Subsequently, a derivative of the Hi-C technique like *in situ* Hi-C was used in rice, sorghum, tomato, and Foxtail millet (Dong et al., 2017; Liu et al., 2017). Other modifications of Hi-C such as Capture Hi-C used in Arabidopsis, digestion-ligation-only Hi-C (DLO Hi-C) in maize (Sun Y. et al., 2020; Nutzmann et al., 2020), and single-cell Hi-C (without biotin purification and pull-down) was used in rice (Zhou et al., 2019).

#### 3.1.1 Chromatin Interaction Analysis by Paired-End Tag (ChIA-PET) Sequencing

Chromatin interaction analysis by paired-end tag (ChIA-PET) sequencing combines chromatin immunoprecipitation (ChIP) with 3C-type analysis for comprehensive and efficient analysis of long-range chromatin contacts bound by a protein like promoters at lower-kilobase resolution (Li et al., 2010; Li et al., 2014). In ChIA-PET, the DNA–protein complex is cross-linked, fragmented by ultrasonication, and captured by the protein-specific antibody. The captured chromatin is attached with a biotin-labeled oligonucleotide linker having a *Mme*I restriction site. The adjacent linkers are connected and *Mme*I restriction enzyme is used to digest the linker to obtain DNA fragments having paired-end tags (PETs). Then PETs are used for high-throughput sequencing (Fullwood et al., 2009). ChIA-PET includes chromatin immunoprecipitation (ChIP) for the enrichment of chromatin interactions, which provides functional specificity and efficiency along with a higher resolution for the detection of chromatin interactions. ChIA-PET generates enriched data for chromatin interaction utilizing the antibody specific to the protein that mediated interactions; hence, it provides a high-resolution map of chromatin interactions with considerably fewer sequencing reads. ChIA-PET also provides abundant unenriched chromatin contact data (similar to that generated in Hi-C) which helps in the plotting of high-order neighborhood/topological proximity. Thus, ChIA-PET provides three different types of genomic datasets for 3D genome analysis: i) the protein binding sites, ii) the enriched chromatin interactions between the binding sites, and iii) unenriched chromatin interactions. A modification in ChIA-PET for long-read was reported with the help of longer paired-end-tags (up to 2 bp × 250 bp) (Li et al., 2017). The longer PET reads improve the mapping efficiency and increase the probability of covering phased single nucleotide polymorphism to enable the identification of haplotype-specific chromatin interactions. While Hi-C is used to identify the spatial/3D proximity (distal interactions) at the genome level (Dixon et al., 2012), ChIA-PET captures the distal interactions involving specific proteins in the genome (Fullwood et al., 2009).

However, depending on the scientific needs, several modifications in Hi-C and ChIA-PET have been adopted. Recent studies using ChIA-PET unraveled chromatin interactions associated with gene expression in maize and rice (Li E. et al., 2019; Peng et al., 2019; Zhao et al., 2019). Maize ChIA-PET studies on chromatin domains with H3K4me3, H3K27ac, and RNA Pol-II identified the network of promoter-enhancer and promoter-promoter interactions in maize. Likewise, the ChIA-PET study on rice revealed the physical interactions between many expression quantitative trait loci (QTL) and target genes (Zhao et al., 2019). These studies present the benefits of identifying/annotating the functional/regulatory chromatin regions/architectures by combining one-dimensional (e.g., epigenetic marks) and 3D genomic (chromatin-chromatin interaction) features.

Hi-C combined with ChIA-PET (chromatin immunoprecipitation) known as Hi-ChIP (Mumbach et al., 2016) was successfully used in maize and wheat (Ricci et al., 2019; Concia et al., 2020). After using biotin to fill in the ends and ligation, the target protein-specific antibody is used to precipitate the DNA–protein complex. Once the specific fragment containing biotin is captured, a transposase-mediated library construction method is used to finally obtain the chromatin conformation bound by the protein of interest. Hi-ChIP requires a very small amount of tissue compared to that required for Hi-C, the signal-to-noise ratio is significantly better, and more informative reads are obtained compared to that obtained from ChIA-PET

## 4 Single-Cell 3D Genomics

Chromatin configuration generally varies in different tissues and cells with changing environmental factors. The chromatin architecture and variations observed by Hi-C/ChIA-PET indicate the population average of cells. Therefore, the chromatin conformation of an individual cell should be captured and studied at the single-cell level. The difference in the 3D genome architecture of cells could be detected by the single-cell 3D genome mapping technique (single-cell Hi-C) (Nagano et al., 2017). Advances in ultra-high resolution microscopy, cytology, and Hi-C provide opportunities to study 3D genome structure at the single-cell level (Wang et al., 2016; Szabo et al., 2018; Sun et al., 2021).

Single-cell 3D genome mapping of mammalian cells demonstrated variation in TADs in different cells, whereas the chromatin compartments and lamina-associated domains remained stable (Stevens et al., 2017). Single-cell chromatin conformation was captured using the Dip-C method by Tan et al. (2018). They could demonstrate the 3D genome architecture of a diploid human lymphoblastoid and a primary blood cell at higher resolution. The cell-specific chromatin organizations like Rabl configuration in mouse embryonic stem cell and Rosette configuration in M/G1phase of the human lymphoblastoid cell line was discovered. Sun et al. (2021) used Hi-C to study 3D chromatin structures in Drosophila cells at different stages of embryogenesis. They observed TAD-like structures in >50% of pre-midblastula transition cells with boundaries at varying locations, while no detectable TAD structure could be observed in the corresponding population Hi-C maps. Although the single-cell 3D genomic study in plants is still in its infancy, it has been performed successfully in rice wherein rice single cell was isolated manually for investigations on chromatin architecture and dynamics during fertilization (Zhou et al., 2019). The study also deciphered the characteristics of chromatin compartments and telomere/centromere at the single-cell level which are distinct from those of mammalian cells (Zhou et al., 2019). Hence, single-cell 3D genomic methods should be further developed and utilized to capture the modulation in chromatin conformation to understand the transcriptional regulation of gene expression at the single-cell level. Application of single-cell 3D genomic analysis in plants would enable a better understanding of the role of chromatin architecture in epigenetic regulation of growth and developmental processes at the cellular (egg, sperm, zygote or a mesophyll cell) level, avoiding the ensemble averaging of folded DNA/chromatin maps prepared for a population of cells.

## 5 Modulation in 3D Genome Architecture

Hierarchical 3D genome organization is observed in yeast, animals, and plants. Higher-order chromatin architectures like CTs and chromatin compartments are fairly conserved among the cell types, tissues, and species (Zheng and Xie, 2019). However, complex modulations in TADs have been observed under environmental changes. TADs were reported to get reorganized rapidly through relocalization of structural proteins from borders of TADs to the interiors in Drosophila in response to heat stress (Li et al., 2015). However, heat shock to human K562 and Drosophila S2 cells caused dramatic transcriptional alterations but no major change in global chromatin architecture was observed (Ray et al., 2019). Therefore, it is necessary to investigate the effects of alterations in chromatin structure on gene expression under varying environmental conditions.

Similar to the compartments and sub-compartments observed in animals (Rao et al., 2014; Rowley et al., 2017), the large global compartment in plants can also be divided into local sub-compartments like heterochromatin, euchromatin, and polycomb (Dong et al., 2017; Liu et al., 2017). The TAD-like domains identified in rice, sorghum, maize, foxtail millet, and tomato could be further divided into four sub-compartments depending on their epigenetic signatures, which include active domain (open chromatin), silenced domain (DNA methylation), Polycomb-repressive domain (H3K27me3 marks), and intermediate type (no specific feature) (Dong et al., 2017). The chromatin-interacting domains (CIDs) identified in rice through long-read ChIA-PET have also been divided into four groups including H3K9me2-associated heterochromatic interacting domains (HIDs), H3K4me3-related active interacting domains (AIDs), RNA polymerase II (RNA-Pol-II)-mediated transcriptional interacting domains (TIDs), and H3K4me3-H3K9me2 mixed interacting domains (Zhao et al., 2019). Similarly, the CIDs identified by the ChIA-PET study possessed distinct genomic features. The AID and TID showed relatively higher expressed gene and active histone mark densities, lower DNA methylation levels, and higher transcription levels. On the contrary, the HID showed the opposite genomic properties. More than half of the TAD-like domains aligned with multiple CIDs, which suggest that the TAD-like domain is a comparatively larger structural unit containing various CIDs. The chromatin regions with similar epigenetic features tether together to form higher-ordered structural units having specific functional consequences (Ouyang et al., 2020).

Plants must perceive and respond to various environmental cues including light, temperature, nutrient status, abiotic and biotic stresses (Kaiserli et al., 2018; Kumar et al., 2021). In response to illumination, the light-inducible loci in Arabidopsis were reported to rapidly change their position from the interior to the periphery of the nucleus (Feng et al., 2014). Such light-induced reorganization of the genome was reported to be associated with transcriptional activation of gene expression. Effects of light on the size of the nucleus, chromatin accessibility, and chromatin organization were reported in Arabidopsis during seedling establishment (Bourbousse et al., 2015). Chromatin interaction maps prepared with *in situ* Hi-C reported stable genome architecture with chromosomal decondensation during cold stress in rice seedlings (Liu et al., 2017). Recently, transposon activation and modulation in the 3D genome of Arabidopsis under heat stress were reported (Sun L. et al., 2020). Increased nuclear size, decreased interactions among KEEs, switching (A→B and B→A) of A/B compartments, and weakening of chromatin compartmentalization under heat stress were demonstrated. However, there is a lack of consensus on modulation in chromatin conformation in response to environmental cues, which need to be built up to better understand the roles of 3D genome organization in gene regulation.

### 5.1 3D Genome Dynamics During Growth and Development

Dynamic changes in 3D genome organization during growth and development are being studied using genome mapping technologies. To better understand 3D genomics and its dynamics over time, a 4D nucleome project in mammals was conceived (Dekker et al., 2017). Several high-order structural reorganizations were observed through chromatin interaction analyses during the development of embryonic stem cells and fertilized eggs in humans (Dixon et al., 2015; Flyamer et al., 2017). However, only a fewer report on the dynamics of 3D genome organization during plant development is available. Changes in chromatin accessibility during plant cell differentiation imply that higher-order chromatin organization is a dynamic process (Wang et al., 2016; Sijacic et al., 2018; Sullivan et al., 2019). The dynamics of 3D genome folding at different developmental stages and growth conditions (4D genomics) in plants need to be explored.

#### 5.1.1 Tissue-Specific Dynamics of Chromatin Architecture

Tissue-specific comparison of 3D chromatin architecture in rice, foxtail millet, and maize using Hi-C revealed stability in global A/B compartments across the tissues with tissue-specific dynamism in local A/B compartments associated with differential gene expression (Dong et al., 2020a). Analysis of mesophyll and endosperm of rice, bundle sheath and mesophyll of foxtail millet, and bundle sheath, mesophyll, and endosperm tissues of maize indicated stable global A/B compartment partitions while dynamic local A/B compartments. Chen et al. (2020) revealed the features of chromatin architecture in sex differentiation in Jatropha, which provides regulatory mechanisms of sex determination in higher plants. Based on the high-quality reference genome assembly prepared with the help of Hi-C data, the differences in chromatin architecture between monoecious and gynoecious floral buds of Jatropha could be identified. The differentially expressed genes (DEGs) were observed to be significantly enriched in altered A/B compartments and TAD regions which occurred preferentially in the differential contact regions between monoecious and gynoecious buds (Chen et al., 2020). The DEGs associated with flower development/hormone synthesis displayed different genomic interaction patterns, which demonstrate that chromatin organization plays important role in the regulation of gene expression during growth and development in plants.

#### 5.1.2 Chromatin Dynamics During Cellular Processes

Chromatin dynamics is not only associated with transcriptional regulation of gene expression but also with other essential cellular processes like DNA replication. The process of DNA replication is essential for genomic content duplication before the cell enters mitosis. DNA replication throughout the genome is generally not a homogeneous process; rather, it is associated with the local histone marks and 3D chromosome architecture. Euchromatin (generally localized in the interior of the nucleus) is replicated earlier than the heterochromatin (localized in the perinuclear region) in animals (Rhind and Gilbert, 2013). Pope et al. (2014) reported that TADs are stable units of replication-timing regulation and replication domain boundaries share a near-perfect correlation with TAD boundaries in humans and mice. Similar studies in Arabidopsis suspension cells reported euchromatin to duplicate early compared to that of heterochromatin (Concia et al., 2018). Moreover, live imaging of replisomes in Arabidopsis revealed dynamics in DNA replication during the S phase of the cell cycle (Yokoyama et al., 2016). The same correlation was observed on comparing chromatin regions with different replication timing in the nuclei of maize root tip indicated open chromatin (euchromatin) to duplicate early compared to the densely packed heterochromatin domains during the S phase (Wear et al., 2017).

Hi-C analysis of tomato and maize genomes showed a large number of long-range chromatin loops to be formed, linking them with interstitial active chromatin regions and suggesting spatial clustering of the expressed genes (Dong et al., 2017). The interaction network of active chromatin by ChIA-PET in maize revealed the role of such physical interactions on gene expression (Li E. et al., 2019; Peng et al., 2019). The formation of chromatin loops connects with active genes, the genes forming long-range chromatin interactions show higher expression, and the gene pairs linked with chromatin loops show co-expression. A recent ChIA-PET study in rice demonstrated coordinated expression of the active genes connected by the formation of chromatin loops (Zhao et al., 2019). These findings suggest that active chromatin domains in nuclei form extensive physical contacts, and associate with gene expression as well as certain essential cellular processes.

### 5.1.3 3D Genome Dynamics During Environmental Stresses

To ensure survival, proper growth, development, and reproduction, plants need to adapt to the prevailing environmental conditions, antedate potential changes, while maintaining the necessary flexibility to respond to other fluctuations. Light, temperature, water, etc. fluctuate on a seasonal basis throughout the year. Long-term adaptation and short-term reaction to environmental factors are underpinned by the changes in gene(s) expression (Franklin et al., 2014). The changes in gene expression and chromatin organization due to histone modifications and nuclear compartmentalization are vital for plant responses to environmental cues (Sun L. et al., 2020; Yung et al., 2021). This section focuses on how environmental factors affect histone modifications, chromatin architecture, nuclear localization, and their effects on regulation of gene expression, plant development, and stress tolerance. These might help to answer some of the questions like: does environmental stress influence positioning/accessibility of gene/chromatin in the nucleus, does such chromatin relocalization relate with the changes in gene expression? These may also help to decipher the structural determinants that energize chromatin localization and chromosomal interactions in cells, tissues, and species in response to the environmental stimuli (4D genomics).

There are two suggested mechanisms, among many other possible strategies, involving different enzymatic paths to accomplish chromatin reorganization. One operates through chromatin remodelers that modulate DNA−histone interactions *via* ATP hydrolysis, while the other utilizes specialized enzymes to (de)methylate DNA or post-translationally modify histone proteins. The SWI2/SNF2 family of chromatin remodeling complexes (part of a large superfamily of helicases−translocases) use ATP energy to gain access to the DNA sequences (Clapier and Cairns, 2009). DNA (de)methylases and histone (de)acetylases [histone acetyltransferases (HATs) and histone deacetylases (HDACs), e.g., BAF60] can regulate the accumulation of methylated DNA base(s) and H3K27me3/H3K9Ac histone marks to control chromatin architecture for regulation of gene expression during the developmental and/or under environmental stresses (4D genomics) (Jegu et al., 2014; Jegu et al., 2017). Despite the remarkable/continuous progress being made in decoding the linear genome, epigenome, and spatial genome architecture (3D genome), regulation of the functional changes in gene expression over time and environmental conditions (4D genome) remains unclear (Aboelnour and Bonev, 2021).

#### 5.1.4 Drought-Induced Chromatin Dynamics

**FIGURE 4:**
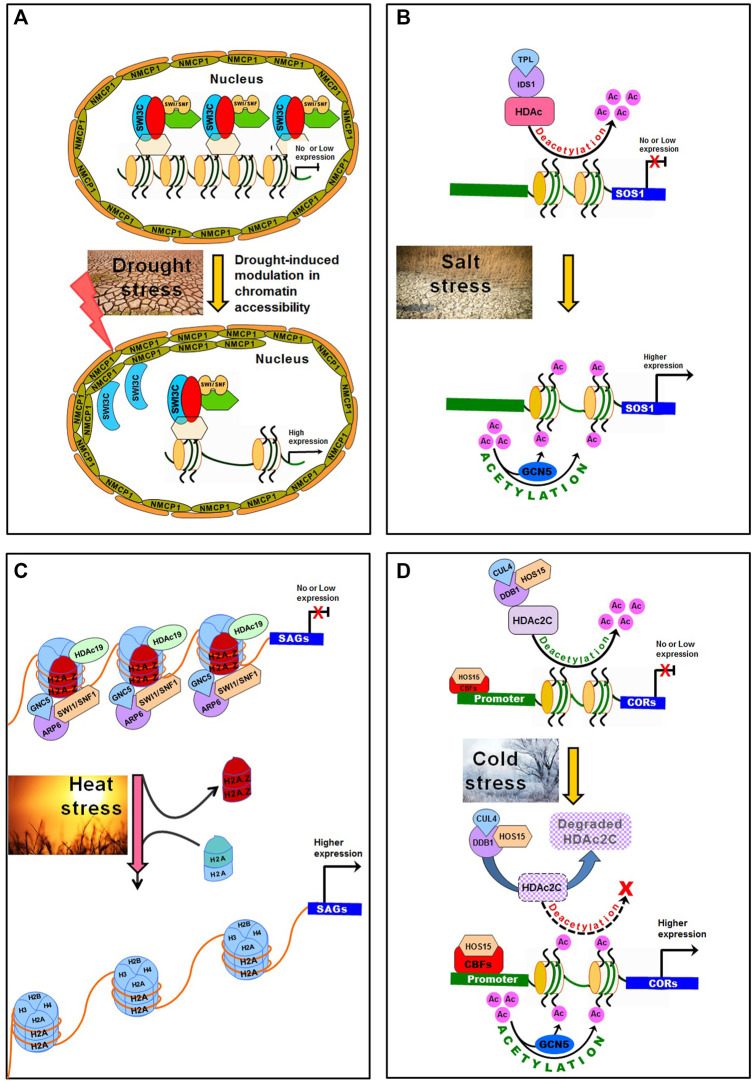
Modulation in chromatin accessibility under abiotic stresses in plants. **(A)** Normally, the lamin-like proteins OsNMCP1 regulate drought tolerance through modulating chromatin accessibility *via* interaction with a chromatin remodeler OsSWI3C in rice. Switch/Sucrose Non-Fermenting (SWI/SNF) complexes interact with OsSWI3C to change the structure of nucleosome, resulting in gene silencing. Under drought stress, OsNMCP1 gets induced and interacts with OsSWI3C, which releases OsSWI3C from the gene-silencing SWI/SNF complexes, resulting in improved chromatin accessibility and higher expression of drought-responsive genes. **(B)** Topless-like/Topless-like protein (TPL/TPR) and Indeterminate Spikelet 1 (IDS1) interact with Histone Deacetylase (HDAc) to form an IDS1-TPL-HDA1 transcriptional repression complex through histone deacetylation. Under salt stress, acetylation of H3K9 and H3K14 by histone acetyltransferase (General Control Non-repressed Protein 5, GCN5), contributes to salt tolerance by activating salt stress-responsive genes (e.g. SOS1). **(C)** Under heat stress, SWI1/SNF1 complex interacts with GCN5 and ARP6 to dissociate H2A.Z (and insertion of H2A into the nucleosome), which causes no transcription of heat-responsive genes. On normal weather, the SWI1/SNF1—ARP6 complex plays important role in placing H2A.Z into the nucleosome. **(D)** Under cold stress, HOS15, in association with DNA Damaged Binding Protein1 (DDB1) and Cullin 4 (CUL4) acts as E3 ubiquitin ligase which degrades HDAc2C causing hyperacetylation of histone H3 on Cold Regulated (COR) chromatin. This makes binding of CBF proteins to COR promoter through High-expression of Osmotically Responsive Gene 15 (HOS15) leading to active expression of COR genes. Moreover, the GCN5 modulates histone acetylation of COR chromatin. At the normal temperature, HOS15 forms a complex with HDAc2C to repress COR expression *via* hypoacetylation of the COR chromatin.

An SWI/SNF chromatin remodeler BAF60 was reported to have dual regulatory functions in epigenomic modification as well as on chromatin architecture. BAF60, having histone deacetylase activity, regulates the level of H3K9Ac histone marks, and transcriptionally suppresses the downstream genes (Jegu et al., 2014; Jegu et al., 2017). The nuclear periphery has a proven role in the regulation of genome topology. Heterochromatic domains were reported to be enriched at the nuclear periphery and Crowded Nuclei 1 (CRWN1) interacts with the chromatin domains in modulating chromatin positioning at the nuclear periphery in Arabidopsis (Bi et al., 2017; Hu et al., 2019). SWI/SNF chromatin remodeler subunit OsSWI3C interacts with OsNMCP1 (a lamin-like protein), which regulates drought tolerance through modulation in chromatin accessibility in rice (Yang et al., 2020) (Figure 4A). Rice possesses a distinct 3D genome pattern of chromosomal compartment folding and spatial distribution which is different from the mammalian 3D genome (Zhao et al., 2019; Zhou et al., 2019). Integration of transcriptome, epigenome and other *omics* data might help better understand the effects of 3D genome dynamics on the regulation of gene expression affecting important agronomic traits, and could lay the foundation for crop improvement.

#### 5.1.5 Salt-Induced Chromatin Dynamics

Chromatin accessibility was reported to be reduced under salt stress in Arabidopsis (Raxwal et al., 2020). Expressions of some of the chromatin remodeling complexes (e.g., SNF2 and SWR1 factors) have been reported to be responsive to salt stress (Li et al., 2011). Chromatin-remodeling complexes are involved in ATP-dependent repositioning of nucleosomes and changes in the core histone composition of a nucleosome, which regulates chromatin accessibility under stressful conditions (Clapier et al., 2017; Yung et al., 2021). Studies also suggest that Topless-like/Topless-like protein (TPL/TPR) interacts with HDAc to regulate stress responses (Tang et al., 2016; Cheng et al., 2018) (Figure 4B). TPL and Indeterminate Spikelet 1 (IDS1) interact with HDAc to form an IDS1-TPL-HDA1 transcriptional repression complex through histone deacetylation. Under salt stress, Pickle (PKL), a well-characterized CHD3-type chromatin-remodeling factor, mediates the accumulation of H3K27me3 at the target gene (Yang et al., 2019). *P*hotoperiod *I*ndependent *E*arly flowering 1 (PIE1) and *A*ctin-*R*elated *P*rotein 6 (ARP6), the components of SWR1 chromatin-remodeling complex (Carter et al., 2018), mediate incorporation of H2A.Z into nucleosomes (Deal et al., 2007). PKL, PIE1, and APR6 were reported to be involved in salt stress tolerance in Arabidopsis (Sura et al., 2017; Yang et al., 2019). As the H2A.Z-enriched nucleosomes are also enriched with H3K27me3 at specific gene loci, PIE1 was suggested to be responsible for the incorporation of H2A.Z into nucleosomes.

Histone deacetylase 1 (OsHDAc1) was reported to repress *OsSOS1* in rice through interacting with a recruiter Indeterminate Spikelet 1 (OsIDS1) (Cheng et al., 2018). In soybean, Plant Homeodomain 5 (GmPHD5) protein (reader of H3K4me2) was reported to interact with HAT and Soybean Imitation Switch (GmISWI) protein (Wu et al., 2011). Acetylation of lysine residues in the tails of histone proteins neutralizes the positive charge and reduces electrostatic interaction between histones−DNA, and helps to loosen the DNA packing, allowing the access of transcription machinery to the gene(s) (Bannister and Kouzarides, 2011). In addition, the chromatin-remodeling factor PKL was reported to modulate chromatin accessibility to other transcriptional regulators, leading to altered expression of salt stress-responsive genes (Yung et al., 2021). The accessibility of a gene was reported to be modulated by post-translational modifications of histone proteins as well as the chromatin-remodelling complexes that regulate nucleosome assembly and spacing (Lusser and Kadonaga, 2003; Hargreaves and Crabtree, 2011; Clapier et al., 2017).

#### 5.1.6 Heat and Light-Induced Chromatin Dynamics

Varying temperature (low or high temperature) significantly affects plant growth and crop yield. Chromatin remodeling is one of the molecular mechanisms implicated in temperature sensing and regulating gene expression (Tasset et al., 2018). Repression of histone deacetylation was reported to prevent hypocotyl elongation under elevated temperatures (Tasset et al., 2018). Exclusion/integration of H2A.Z nucleosomes has been another chromatin remodeler that increases chromatin accessibility leading to changes in gene expression under elevated temperature (Quint et al., 2016; Cortijo et al., 2017; Dai et al., 2017) (Figure 4C). The increased temperature was reported to induce H3K9 deacetylation of nucleosome of PIF4 and YUCCA8 loci (Tasset et al., 2018; van der Woude et al., 2019) which are involved in temperature responses (Franklin et al., 2014; Quint et al., 2016). In many higher eukaryotes, heterochromatin comprises transposable elements (TEs) which are silenced by epigenetic modifications. Hi-C analysis for comparative genome-wide high-resolution chromatin packing under normal and heat stress conditions, the stress was reported to cause global rearrangement of 3D genome in Arabidopsis. Heat activation of TEs correlates with reduced chromosomal interactions engaging pericentromeric, KNOT, knob, and upstream and downstream flanking regions of activated TEs (Grob et al., 2014).

Temperature and light influence the developmental trajectory/morphology of the plant. The light-regulated modulations in chromatin architecture were initially reported based on the photomorphogenesis responses (Barneche et al., 2014). Studies show that shifting from dark to light results in increased nuclear size and the number of chromocenters in Arabidopsis. Changes in chromatin architecture and nuclear organization can modulate gene expression, which leads to short- and long-term plant acclimatization/adaptation to the environment. Hence, it becomes important to investigate the changes in chromatin architecture (composition, structure, and topology) that modulate the expression of genes in response to the variations in temperature and light (4D genomics).

#### 5.1.7 Cold Stress-Induced Chromatin Dynamics

In plants, exposure to cold stress has been reported to alter chromatin configuration through the autonomous pathway and silencing of the MADS-box transcriptional repressor of Flowering Locus C (FLC) (Fornara et al., 2010; Wu et al., 2019). When the temperature returns to normal, FLC is silenced and activates the flowering genes (Fornara et al., 2010). Other proteins, like Curly Leaf (CLF) and Swinger (SWN) having H3K27me3 activity, mediate *FLC* repression during the vegetative stage of plant development (Bouyer et al., 2011; Lopez-Vernaza et al., 2012). *Clf* mutants were reported to show reduced H3K27me3 repressive mark causing up-regulated expression of *FLC* (Lopez-Vernaza et al., 2012). Some of the members of Polycomb repressive complex 2, which constitute CLF and SWN proteins, are also required for silencing of *FLC* (Berry et al., 2017; Portoso et al., 2017; Laugesen et al., 2019). When the *FLC* chromatin is active, it shows a low level of H3K27me3, and a high level of histone marks (H3K4me3, H3Ac, and H3K36me3) associated with transcriptionally active chromatin (Hyun et al., 2017; Wu et al., 2019). A recent study reported *H*igh-expression *O*smotically *R*esponsive gene 15 (HOS15) to work together with Histone Deacetylase 2C (HDAc2C) by directly binding to Cold Regulated (*COR,* e.g., *COR47* and *COR15A*) genes’ promoter (Park et al., 2018). Histone acetylation/deacetylation (by HAT and HDAc) was reported to play role in cold responses (Kim et al., 2015). Arabidopsis histone deacetylase (HDAc6) was reported to be up-regulated by cold stress to positively regulate cold tolerance (To et al., 2011). Under normal temperature, HOS15 and HDAc2C make a complex that represses the expression of *COR* genes by hypoacetylation at the chromatin. Under cold stress, HOS15 acts as an E3 ubiquitin ligase in association with *D*NA *D*amaged *B*inding protein 1 (DDB1) and Cullin 4 (CUL4) to degrade HDAc2C, which leads to hyperacetylation of histone H3 at *COR* chromatin. This promotes CBF proteins binding at the *COR* promoters *via* HOS15 to activate *COR* genes expression. Moreover, the GCN5 promotes H3 acetylation at *COR* genes (Ding et al., 2019) (Figure 4D).

Although compartmentalization of genome into territories, compartments, TADs, and loops appears to arise largely independent of each other, the layers of genome folding is redundant, at least partially, which help maintain the gene expression pattern (Aboelnour and Bonev, 2021). Chromatin loops are highly context-dependent and rely on the *cis*-acting elements as well as on the local chromatin environment to coordinate gene expression in a time environment dependant manner. How the regulatory loops are established and remodeled during the developmental processes and environmental stresses, and what is the functional importance of physical proximity with the changes in linear epigenome are some of the critical questions in the field of 4D genomics. Thus, genome architecture is highly diverse across the cells, tissues, and species suggesting that the relationship between 3D genome organization and molecular events like transcription/gene expression is highly dynamic.

## 6 Difference in Plant and Animal Chromatin Organization

It is well-established now that spatial organization of chromatin plays important roles in several biological processes like DNA replication, repair, gene expression, repression of TE, etc. Therefore, investigations on the 3D organization of chromatin architecture would enable a better understanding of the transcriptional regulation of gene expression/biological process. Recent advances in NGS-based 3C technologies have enabled us to examine the 3D organization of chromatin at unprecedented scale and resolution. 3D genome organizational studies indicate conserved but distinct chromatin structures between mammals and plants at different scales ranging from chromatin loops to chromosome territories (Dogan and Liu, 2018). Chromatin organization in mammals could be presented mainly at three hierarchical levels: compartments, domains, and loops that play important roles in the transcriptional regulation of genes. Though similar organizational levels have been reported in plants, these may not have the same functions as they have in their mammalian counterpart. Combinations of 3C and high-throughput sequencing techniques have considerably improved our understanding of the spatial organization of chromosomes. While Hi-C captures all the chromatin interactions at low resolution (Lieberman-Aiden et al., 2009), ChIA-PET (Fullwood et al., 2009) and Hi-ChIP or PLAC-Seq (Fang et al., 2016; Mumbach et al., 2016) generate high-resolution interaction maps of the loci occupied by proteins (modified histones, transcription factors, and RNA polymerase II) which can be pulled down by ChIP. These techniques provide extraordinary insights into 3D chromatin architecture and functions, but only a little is known about the functions of the chromatin structural organization in plants.

Studies show that active chromatin interacts with other active regions, and repressive chromatin interacts with other repressed regions. Thus, a genome is partitioned into two different compartments: active/euchromatin and repressive/heterochromatin, which are referred to as A and B compartments, respectively. Mammalian A compartment is actively transcribed, open chromatin, enriched with active histone marks like H3K4me3 and H3K27ac having high GC content. On the other hand, the B compartment is enriched with repressive histone marks like H3K9me3, associated with the nuclear lamina, and rich in AT (Lieberman-Aiden et al., 2009; Ryba et al., 2010). The compartment partitioning is dynamic and switches frequently in different tissues or at developmental stages. Dixon et al. (2015) reported that 36% of the human genome switches for the compartments and the loci that switched from A to B showed decreased expression, while those switched from B to A showed increased expression. In plants, the actively transcribed euchromatin arms form the A compartment and the pericentromeric heterochromatin forms the B compartment (Feng et al., 2014; Grob et al., 2014). This partitioning is largely stable across tissues (Dong et al., 2020b), and reduced compartment interaction has been reported in DNA methylation mutants of Arabidopsis (Feng et al., 2014), and in the endosperm tissues of rice/maize where DNA demethylation occurs naturally (Dong et al., 2020b). Based on Hi-C interaction analysis of Arabidopsis chromosome arms, the regions observed to interact with chromocenter were named *C*ompacted *S*tructural *D*omains (CSDs), while the other regions containing active/expressed genes are called *L*oose *S*tructural *D*omains (LSDs) (Grob et al., 2014). Moreover, CSDs are associated with the nuclear periphery and require lamina-like proteins (CRWN1 and CRWN4), as well as DNA methylation at CHG and CHH contexts (Bi et al., 2017; Grob and Grossniklaus, 2019; Hu et al., 2019).

**FIGURE 5:**
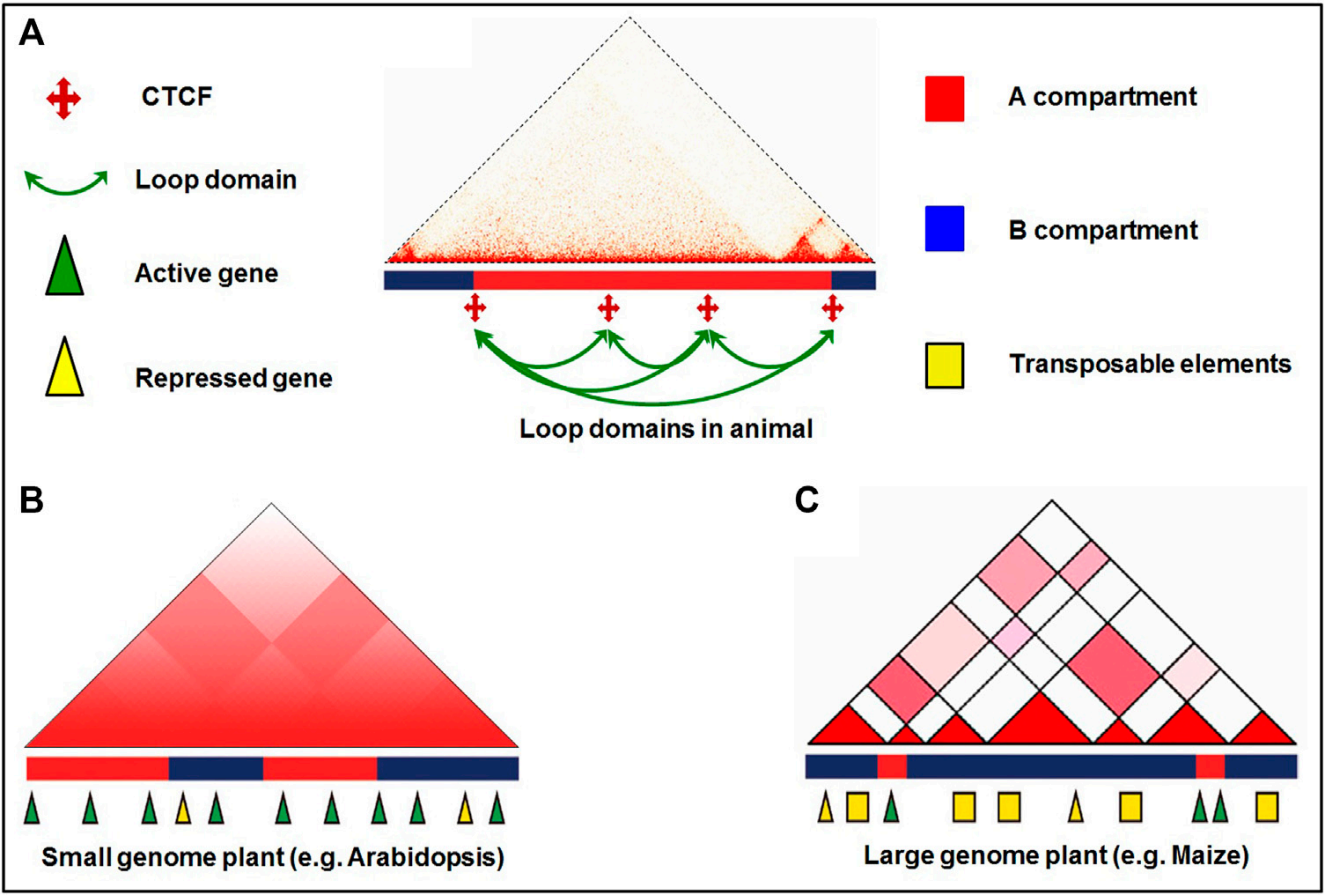
Comparison of 3D chromatin organization of animals and plants. **(A)** Loop domain in the mammalian genome. CTCT loops are formed at the domain corner, and these domains are located within a compartment. Small adjacent loop domains form larger domains having nested structures. The dynamics of a loop domain is associated with changes in CTCF binding. **(B)** In Arabidopsis, chromosome arms are partitioned into loose structural domains (LSDs) and compacted structural domains (CSDs) which are comparable to the local A/B compartments rather than the mammalian TAD and the global compartment domain of large-genome plant. **(C)** Compartment domains in the large-genome plants often overlap with local compartments having active genes located inside the domain associated with the A compartment. Transposable elements and repressed genes are located in the domain with the B compartment.

Chromatin domains are a prominent feature in the mammalian genome and are referred to as TADs. Interaction frequency within a TAD is higher than that between TADs, which reduce significantly at the domain boundaries (Dixon et al., 2012; Nora et al., 2012). Borders of some TADs were reported to have CTCF and cohesin to help the formation of chromatin loop, known as “loop domain” (Figure 5A) (Rao et al., 2014). Cohesin binding has been reported to be highly mobile, and its binding often occurs at the inner side of CTCF at the TAD border in the human genome (Tang et al., 2015). Flipping of CTCF binding site disrupts the TAD (Guo et al., 2015), and degradation of CTCF/cohesin subunit also disrupts the TAD structure (Nora et al., 2017; Rao et al., 2017). Based on high-resolution Hi-C analysis, human TADs could be further partitioned into subdomains (subTADs or contact domains) (Rao et al., 2014). Although TAD is not a prominent feature in Arabidopsis (Feng et al., 2014; Grob et al., 2014), TAD-like structures could be identified in Arabidopsis wherein boundaries are enriched with active genes/active epigenetic marks like open chromatin, H3K4me3, and H3K9ac (Wang et al., 2015). Compared to the mammalian TADs, Arabidopsis TAD-like structures are smaller and the interaction is weaker (Figure 5B). The occurrence of a few TAD-like structures was also reported in H3K27me3-rich and H3K9me2-rich chromocenter heterochromatic regions in Arabidopsis (Feng et al., 2014; Rowley et al., 2017). However, in plants having larger genomes like maize and tomato more frequent occurrence of TAD-like structures could be identified (Figure 5C) (Dong et al., 2017; Liu et al., 2017; Wang Q. et al., 2018; Dong et al., 2018; Dong et al., 2020a).

Distal regulatory elements can interact physically with genes through the formation of loops, which is well-studied in the β-globin gene revealing a causal relationship between looping and gene activation (Smallwood and Ren, 2013). The most prominent loops are observed between the loci bound by the CTCF and cohesion, which show higher interaction frequency and are relatively conserved (Dowen et al*.*, 2014). Gene-to-gene and gene-to-distal active chromatin loops were recently identified in rice and maize (Ricci et al., 2019; Zhao et al., 2019). Such chromatin loops are often observed for the expression of QTL (Peng et al., 2019). Unlike the loops observed in animals, plant domains are not enriched with the loops 10 Kb range, which support the argument that plant domains do not confine enhancer-promoter interactions, in contrast to the mammalian TADs. The distance between two loci joined by a chromatin loop in maize was observed to be shorter for the syntenic gene pairs in the related species like rice, sorghum, and millet compared with those of the non-loop genes (Dong et al., 2020a). Despite a huge variation in genome size, most of the plant species have similar numbers of genes/open chromatin regions but due to the insertion of TEs and repeats between genes and distal regulatory elements the genome size increases (Dong et al., 2020b).

## 7 Constraints of 3D Genomics Techniques

The discoveries made with the use of 3D genomics techniques including hierarchical chromatin structures like chromatin loops, TADs, A/B compartments and sub-compartments, chromatin territories have revolutionized structural and functional genomic analyses. However, there are certain limitations of these techniques. Most of the 3D genomics analyses indicate chromatin configuration of population average which varies in different tissues/cells with changing environmental conditions. Therefore, the need of the day is to study chromatin conformation at the single-cell level. Though FISH provides easy visualization of repetitive sequences and dynamics at an individual locus (Cui et al., 2016), its stringent preparation/protocol affects chromatin organization. Similarly, low throughput coverage of 3C, limitation of 4C to one viewpoint, and limited coverage of 5C are some of the constraints being faced by the researchers. Moreover, 5C may not be suitable for interaction studies on relatively smaller genomes like that of yeast, Drosophila, and Arabidopsis (Zhang and Wang, 2021). Similarly, Hi-C can be better used for studying alterations in TAD/supra-TAD in chromatin organization, but it may not be appropriate for studies on the individual locus (Sati and Cavalli, 2017). As Hi-C relies on RE to break chromatin into smaller fragments, the restriction/recognition sites are heterogeneously distributed in the genome which limits the spatial resolution of the contact map (Ma et al., 2015). Incomplete digestion by RE, spurious ligation, and cross-molecular ligation (noise) might perplex the Hi-C findings (Hoshino et al., 2017). Different experimental methods result in the identification of different TAD sizes and numbers (Zufferey et al., 2018) probably because of the low coverage of the 3C/derived techniques (Xu et al., 2020) and the different models that each algorithm employs (Boltsis et al., 2021). In single-cell Hi-C analysis, detection of TADs is generally not reproducible but reassembled on combining the maps for a population (Flyamer et al., 2017). This strengthens the view that a TAD is visible only when many cells are analyzed. However, further optimization and advances in the techniques with increased resolution and coverage are expected to make 3D genomics/Hi-C an exciting discovery.

## 8 Future Perspectives

Chromatin conformation has considerable effects on gene expression and regulation, and regions with strong chromatin interaction generally show functional dependency (Mendes et al., 2013). Alterations in chromatin compactness affect the accessibility of chromatin to TFs, chromatin remodelers, and transcriptional machinery, which influence gene expression levels (Rutowicz et al., 2019). Single-gene resolution Hi-C map of Arabidopsis showed that local chromatin loops (between the 5′ and 3′ ends of the genes) were associated with highly expressed genes (Liu C. et al., 2016). ChIA-PET and DLO Hi-C based high-resolution chromatin interaction maps of maize demonstrated chromatin loops to be formed between the regulatory elements and the genes (Li E. et al., 2019; Peng et al., 2019; Sun Y. et al., 2020). Promoter–promoter interaction map associated with H3K4me3 and RNA polymerase II in rice reported co-transcription of the genes (Zhao et al., 2019). Based on the DNA methylation, histone modification, and chromatin accessibility data, enhancers are being identified, which are noncoding DNA elements that function independently of transcriptional direction, relative position with the promoter, and participate in gene regulation through long-distance chromatin interaction through chromatin loop formation (Zhao et al., 2019).

Until recently, studies on 3D genome organization have been challenging tasks due to technical difficulties; however, technological advances have enabled us to take up such studies with unprecedented resolution and accessibility. Technological developments in the 3D genomics techniques like ChIA-PET (Fullwood et al., 2009), Capture-Hi-C (Mifsud et al., 2015), and Hi-ChIP (Mumbach et al., 2016) have enabled investigating short- and long-range chromatin interactions with better resolution for their regulatory roles (Li E. et al., 2019; Ricci et al., 2019; Concia et al., 2020). With the integration of robotics and microfluidics, 3D chromatin topology can be analyzed at a single-cell level for cell-type-specific studies (Boettiger and Murphy, 2020). Moreover, the resolution limit imposed by traditional microscopy can be surmounted by next-generation, super-resolution (optical resolution ≥50 nm) techniques like structured illumination microscopy (SIM), photoactivated localization microscopy (PALM), stochastic optical reconstruction microscopy (STORM), and stimulated emission depletion microscopy (STED) (Schubert, 2017; Xu and Dixon, 2020). Such next-generation microscopy for visualizing chromatin architecture has been used in mammals Xu and Dixon, 2020); however, these have rarely been used for plant studies. While 3C-based techniques provide a high-resolution map of chromatin state/genomic region of interest, the next-generation super-resolution microscopy complements the techniques by providing nano-scale imaging. Imaging resolution can be further improved by using two/multiphoton microscopy, which allows fast and dynamic imaging of nuclei (Komis et al., 2018).

Fine structures of the 3D genome are also being investigated by combining improved CRISPR technologies with ultra-high resolution microscopy in mammals. Locations of transcriptionally active and inactive regions in the nucleus were determined using sgRNAs (targeting 16 MS2 binding motifs) and catalytically inactive Cas9 (dCas9) protein (Qin et al., 2017). Moreover, CRISPR technology was also used for the functional validation of 3D genome folding by knock-out/knock-in of TAD boundary/structural proteins (CTCF and cohesins) involved in chromatin loop formation in animals (Guo et al., 2015; Lupianez et al., 2015; Fei et al., 2019). Knocking-out of 3D structural elements using CRISPR technology, TAD, and loop structures could be altered in plants which affected gene expression (Pei et al., 2021). TAD boundaries in rice and maize exhibited enrichment of plant-specific transcriptional factor binding sites, which indicates the possibility of TFs being involved in the formation of TADs (Liu et al., 2017), as observed in mammals (Stadhouders et al., 2019). Enhancers can also be knocked out to explore their effects on gene expression, which may prove to be an efficient technique for functional validation of 3D genomic findings. The lack of CTCF homologs, but the presence of cohesin homolog subunits, and TADs being not as distinct in plants as in animals (Liu et al., 2002; Zhang et al., 2020) would require further investigations on plant 3D genomics.

Certainly, there is still a lot to examine and learn, which would require more contact maps at higher resolution particularly for the plant genomes differing in size and gene density. To gain more knowledge, a comparison of the contact maps of the same genome under different environmental conditions and/or developmental stages (4D genomics) of a single cell or single cell type would be desirable. Moreover, the use of synchronized cells would help to understand the changes in chromatin architecture during the cell cycle. Furthermore, the participation of RNAs in the formation/maintenance of chromatin structures, if any, would also need to be studied. Finally, several outstanding questions will need to be answered including, but not limited to: i) do different chromatin structures exist in a cell type under changing environmental conditions and/or developmental stages? ii) Do TADs/TAD-like structures exist only in the nuclei of plant species with larger genome sizes? iii) Can the changes in gene expression modify chromatin configuration? As soon as we would get answers to these questions, several other new questions will be required to be answered. Indeed, the experiments designed to answer some of these questions are on the go in laboratories worldwide, and we believe that the next 5 years of research on 3D genomics would be more exciting than they had been in the past.

## 9 Conclusion

Sequencing and assembly of genomes for model animal and plant species were some of the ground-breaking biological research findings of the second half of the 20th century. After preparing the draft genome for the model organisms, the scientific attention moved to annotate and decipher the biological functions of protein-coding genes to get the answer to many relevant biological questions. After understanding the biological function of specific gene/protein and protein complexes, which has provided better understanding in all the fields of biology, now it has become clear that the DNA/genome sequence itself is not the absolute determinant of phenotypic traits. Subsequently, researchers around the world started investigating the so-called ‘junk DNA’ (which in some cases embodies the vast majority of the eukaryotic genome) that might play regulatory roles in gene expression. Hence, during the last 3 decades, efforts were made to explore the non-genetic/epigenetic/3D genomic mechanisms/features responsible for phenotypic plasticity observed in living beings.

With the advances in 3D genomics technologies, chromatin loops are being detected with the help of Hi-C/ChIA-PET which identify enhancer−promoter interactions affecting gene expression (Liu C. et al., 2016; Wang M. et al., 2018; Li E. et al., 2019; Peng et al., 2019; Sun Y. et al., 2020). Moreover, the effects of genetic structural variation (SV) on chromatin organization in rice were analyzed which revealed alterations in chromatin topology and the rate of transcription (Zhao et al., 2019). Sequence variation and meiotic recombination rate were reported to correlate with 3D genome structures. TADs showed more single nucleotide polymorphism, SVs, and higher recombination compared to that in the inter-TAD regions, which could be associated with the epigenetic landscape of TAD, TE composition, and increased incidence of meiotic crossovers (Liao et al., 2021). Implementation of state-of-the-art techniques like CRISPR/dCas9 for editing the interacting regions/regulatory elements and chromatin interactions with the help of RNA molecules can be of particular interest to better understand the regulatory functions of chromatin architecture. Understanding the spatial organization of the genome in the nucleus and their functional implications have become a fundamental pursuit in the post-genomic era (Kong and Zhang, 2019), as this allows integration of the knowledge of linear genome with epigenomic/3D genomic regulatory networks and phenotypic data. Future 3D genomic studies will greatly benefit from the investigations at the single-cell level with the help of advancing long-read sequencing techniques and live-cell imaging which would be the key to deciphering the importance of 4D genomics for manipulation of gene regulation/expression towards the development of climate-smart crops.
